# Novel cyclic C_5_-curcuminoids possess anticancer activities against HeLa cervix carcinoma, HEC-1A adenocarcinoma, and T24 bladder carcinoma cells

**DOI:** 10.1186/s12935-025-04077-2

**Published:** 2025-12-03

**Authors:** Edina Pandur, Petra Schenk, Győző Kulcsár, Gergely Gulyás-Fekete, Katalin Sipos, Imre Huber

**Affiliations:** 1 Department of Pharmaceutical Biology, Faculty of Pharmacy, University of Pécs, Rókus Str. 4, Pécs, H-7624 Hungary; 2 Institute of Pharmaceutical Chemistry, Faculty of Pharmacy, University of Pécs, Rókus Str. 4, Pécs, H-7624 Hungary; 3 Department of Biochemistry and Medical Chemistry, Medical School, University of Pécs, Szigeti Str. 12., H-7624 Pécs, Hungary

**Keywords:** Carcinomas, Cyclic C_5_-curcuminoids, Ferroptosis, Apoptosis, Anticancer activities

## Abstract

**Background:**

Umin was discovered in *Curcuma longa* L. and/or *Curcuma domestica* L. C_5_-curcumin and its derivatives, like synthetic cyclic C_5_-curcuminoids, are promising anticancer compounds with exceptional pharmacokinetic profiles compared to curcumin.

**Methods:**

To demonstrate their anticancer activities, we tested six novel synthetic cyclic C_5_-curcuminoids on HeLa, HEC-1A, and T24 tumor cell lines. This investigation focused on ferroptosis and apoptosis, two types of programmed cell death. Ferroptosis-related genes were investigated using real-time polymerase chain reaction (PCR) and Western blotting. The total iron content, reactive oxygen species (ROS) levels, glutathione peroxidase activity, and thiol concentrations were measured to determine ferroptosis. Cytochrome c levels and caspase-3 activity were measured to monitor the apoptosis.

**Results:**

The study of six synthetic cyclic C_5_-curcuminoids revealed that their effects on HeLa cells differed from those on HEC-1A and T24 cells, indicating distinct mechanisms of action. Compound 4 notably increased iron accumulation and reactive oxygen species (ROS) production, while decreasing antioxidant defenses in all three carcinoma cell lines, suggesting a ferroptotic response. In contrast, compound 9 was successful in activating caspase-3 in carcinoma cells and inducing apoptosis in the COS-1 control cell line. Notably, compound 4 did not enhance caspase-3 activity in the control cell line. These results highlight compound 4 as a possible synthetic cyclic C_5_-curcuminoid for the three cancerous cell lines tested.

**Conclusion:**

Regarding the distinct effects of the examined synthetic cyclic C_5_-curcuminoids on the three cancer cell types, we hypothesize that their mechanisms of action are different and that divergent target molecules and/or signaling pathways may be involved. However, compounds 4 and 9 were efficient against the three carcinoma cell lines. Further examination of the possible targets could help elucidate which compounds are more suitable for consideration as potential antitumor drug candidates.

**Supplementary Information:**

The online version contains supplementary material available at 10.1186/s12935-025-04077-2.

## Background

Despite the enormous progress made in this field, cancer remains a major health problem worldwide. According to WHO reports, owing to key facts (e.g., cancer mortality is nearly one in six deaths worldwide, with a growing prevalence), successful treatment remains a challenge in this century [1]. Therefore, international research on new and more active drug candidates for cancer is fundamental. One of the most intensely cultivated pharmaceutical research areas is the examination of analogs of natural compounds found in plants to discover promising model molecules [2].

**Fig. 1:**
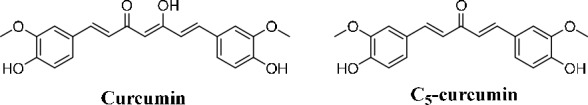
Structures of the two natural compounds, curcumin and its analog, C_5_-curcumin

The long history of curcumin as a nutraceutical and later as a pharmaceutical is an example of this. The therapeutic capacity and constraints of curcumin have been extensively reviewed [3, 4]. C_5_-curcumin, a natural model molecule of one of the most important groups of curcuminoids, cyclic C_5_-curcuminoids, was discovered in 1993 in the same family of plants isolated from Curcuma longa L. and/or Curcuma domestica L. (Fig. 1).

The group of C_5_-curcuminoids is derived from C_5_-curcumin, which serves as a truncated analog of curcumin (1,5-bis(4-hydroxy-3-methoxyphenyl)−1,4-pentadiene-3-one), in which there are only five carbon atoms in the spacer between the two aromatic rings. However, one family of curcuminoids can be directly derived from the structure of curcumin. These are the so-called C_7_-curcuminoids (1,7-bis(4-hydroxy-3-methoxyphenyl)−1,6-heptadien-3,5-dione), which contain seven carbons in their *ß*-dienedione spacer [5].

C_5_-curcumin and its derivatives exhibit better pharmacokinetic behavior than curcumin [6, 7]. Numerous publications over the past three decades have reported their synthesis and exceptional anticancer potential against diverse tumor cell lines [8–10].

Curcumin-containing products are currently being examined in clinical trials for various tumors, such as multiple myeloma, liver and intestinal cancers, and head and neck cancers [11–14]. Curcumin has been investigated as a supplementary therapy for antitumor therapies with promising results [15–18]. Data obtained from clinical studies suggest a possible antitumor effect of curcumin-containing products [19–25].

**Fig. 2:**
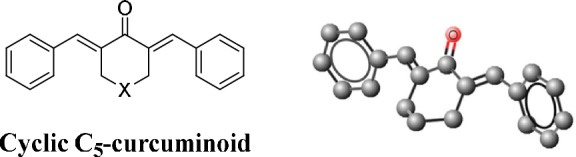
The cross-conjugated general structure of cyclic C_5_-curcuminoids with two benzylidene double bonds (X can be CH_2_, oxygen, sulfur, NH, substituted N, CHOH, or CHOR). The primary binding sites of the molecules are the thia-Michael acceptor benzylidene double bonds. The auxiliary binding moiety (secondary binding site) is X, which is located in the central position

The stereochemical structures of cyclic C_5_-curcuminoids (Fig. 2) are fixed in a close-to-planar shape, with minimal conformational movements compared to curcumin. Ten atoms located in the central ring and the entire *β-dienone* motif are in one plane (primary binding site). The only atom in the central ring outside this plane is X (the secondary binding site). The free rotation of the two aromatic rings is also hindered. Cyclic C_5_-curcuminoids exhibit decreased conformational activity, which seems to be significant for enhancing their pharmacological efficacy relative to curcumin.

From both chemical and pharmacological perspectives, it is crucial to underline the highly specific reaction of these C_5_-curcuminoids, namely, their thia-Michael reaction: their benzylidene double bonds (primary binding site) do not react with amino or hydroxyl groups, either chemically or biochemically. In other words, benzylidene double bonds are the only thiol acceptors. They can react with more than one thiol group in different biological peptides, providing a good biochemical tool for multiple targets in their biological places of action [14].

This may also account for their ability to reverse multidrug resistance (MDR) in antibacterial and anticancer treatments [17–20, 26]. In vivo data have also shown good oral tolerability and the absence of acute toxicity of this cyclic C_5_-curcuminoid family of compounds in rodents [21, 27].

**Fig. 3:**
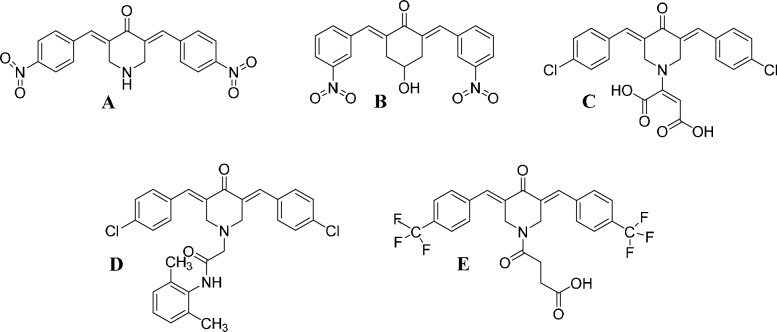
Previously tested anticancer cyclic C_5_-curcuminoids (**A**–**E**) with different aromatic substituents on the primary binding *ß*-dienone moiety and various X motifs in the central ring were used as auxiliary binding tools

Our laboratory primarily focuses on the synthesis and anticancer evaluation of novel cyclic C_5_-curcuminoids. We found that compound **A** (Fig. 3) is approximately 80 times more active as an antiproliferative agent against human non-invasive breast adenocarcinoma (MCF-7) than curcumin [28].

Recently, we reported the synthesis of a novel class of cyclic C_5_-curcuminoids with a 4-hydroxylic function on the central ring of the molecule. Derivative **B** (Fig. 3) with an *m*-nitro aryl substituent exhibited the highest activity against human A2780 (ovarian), C33A (cervix), and MDA-MB-231 (breast) tumor cell lines [29]. Derivatives **C**, **D,** and **E** possess different N-substituents in the central ring (auxiliary binding function) (Fig. 2). Compound **C** exhibited remarkable antiproliferative activity against four human adherent cancer cell lines, HeLa, A431, A2780, and MCF7, in a direct comparison study of mono- and benzylidene *ß*-dienone derivatives [30].

Compounds **D** and **E**, along with 21 other newly synthesized molecules, were evaluated for their efficacy against human neuroblastoma (SH-SY5Y) and human grade IV astrocytoma (CCF-STTG1) cell lines at low concentrations ranging from approximately 0.2 nM to 8 nM. These compounds exhibited significant proliferation-inhibitory activity, with IC50 values in the low nanomolar to picomolar range. They exhibit a certain selectivity (SI = 5–6) and ability to penetrate the blood–brain barrier (BBB), depending on their albumin-binding characteristics [31].

Building upon our previous research, we aimed to comprehensively investigate how the structure of the secondary binding part influences the antitumor properties of our novel cyclic C_5_-curcuminoids, which feature new substituents on the central ring, in carcinoma cell lines. Carcinoma is the most prevalent form of cancer, comprising 80–90% of all cancerous diseases. Despite extensive efforts dedicated to cancer research to find new and efficient treatments, numerous cancer types have high mortality rates because they are difficult to treat [32–34].

In this study, we focused on bladder carcinoma (T24) and endometrial adenocarcinoma (HEC-1A), where the effects of curcumin have been studied, but not those of curcumin analogs and cyclic C_5_-curcuminoids. In the HeLa cell line, we were interested in determining whether our six novel cyclic C_5_-curcuminoids exhibited lower IC50 values and higher efficacy than the derivatives previously tested in the literature [35–40]. Therefore, we treated the three carcinoma cell lines with the new synthetic C_5_-cyclic curcuminoids to investigate their potential anticancer effects. Based on these findings, we propose that the examined curcuminoids activate iron-mediated apoptosis (ferroptosis) by elevating iron content, ROS production, and lipid peroxidation, and decreasing antioxidant activity. Moreover, they triggered apoptosis by stimulating caspase-3 activity. However, the mechanisms by which these cyclic C_5_-curcuminoids exert their effects on various cancers are not uniform, supporting the observation that curcuminoids act via different molecular targets.

## Materials and methods

### Chemistry

All chemicals, solvents, and reagents used in the experiments were purchased from Alfa Aesar Ltd. (Budapest, Hungary), Molar Chemicals Ltd. (Halásztelek, Hungary) and Merck Life Sciences Ltd. (Budapest, Hungary). A Barnstead-Electrothermal 9100 apparatus was used to ascertain the uncorrected melting points. Silica gel 60 (0.2–0.5 mm, Merck Life Sciences Ltd, Budapest, Hungary) was used for column chromatography, and pre-coated silica gel 60 (F-254, Merck Life Sciences Ltd, Budapest, Hungary) plates for thin-layer chromatography (TLC).

The nuclear magnetic resonance (NMR) ^1^H- and ^13^C-spectra were recorded using a Bruker Avance III 500 (500.15/125.77 MHz for 1H/13C) spectrometer, as described in our previous publication [31]. The chemical shifts were calibrated against the residual solvent signals. The measurements were conducted at a probe temperature of 298 K using a dimethyl sulfoxide (DMSO)-D_6_ solution. The NMR spectra are presented in Additional File 1.

A Thermo Dionex Ultimate 3000 liquid chromatograph (Dionex, Sunnyvale, CS, USA) coupled with a Thermo Q Exactive Focus hybrid Quadrupole-Orbitrap mass spectrometer (MS) (Thermo Fisher Scientific, Waltham, MA, USA) was used for HPLC–MS analyses, as described in our previous publication [29]. For data analysis and evaluation, Q Exactive Focus 2.1 Software and Xcalibur 4.2. Software (Thermo Fisher Scientific Inc., Waltham, MA, USA) were applied. The separation of the compounds was performed on a Thermo Hypersil GOLD C18 analytical column. The mobile phase consisted of 0.1% formic acid solution (A) and methanol containing 0.1% formic acid (B). Gradient elution was performed as follows: 0 min, 20% B; 6 min, 80% B; 8 min, 80% B; 10 min, 20% B; and 10 min stop. Chromatographic analysis was conducted at 40 °C with a flow rate of 0.3 mL/min. The injection volume was 5 μL [29].

Mass spectrometry analyses were conducted utilizing switched ion mode, maintaining consistent parameters. The electrospray ionization (ESI) source was operated at a spray voltage of 3.50 kV, and the capillary temperature was set to 350 °C. The sheath gas was delivered at 30 AU and maintained at 450 °C. Aux gas was delivered at a rate of 10 AU. Full-range mass spectra (100–1500 m/z) were acquired under optimal conditions [29]. The MS spectra are presented in Additional File 2. The physical and analytical characteristics of the resynthesized compounds **1–3** were consistent with those of the original compounds [28, 29, 41].

### Synthesis of cyclic C_5_-curcuminoids 4–9: experimental conditions and spectral data

#### 1-(2’-Thiazolin-2’-yl)-[(*3E,5E*)−3,5-bis(p-methoxybenzylidene)]-piperid-4-one (4)

Compound **1** (2 mmol) was dissolved in a 25 mL chloroform–methanol (1:1) mixture containing 2-chloroethyl isothiocyanate (2 mmol) and triethylamine (0.40 mL). This solution was stirred at room temperature till the spot of 1 disappeared from the TLC plate (approximately four hours). After the addition of 10 ml of water, the water phase was extracted with a further 2 × 10 ml of chloroform. Combined chloroform phase dried (Na_2_SO_4_ sicc), filtered, and evaporated. The crude product was a yellow crystalline material. Yield 90%. Mp.: 200–201 ºC after recrystallization from chloroform/methanol. ^1^H-NMR (500 MHz, DMSO-D_6_) δ (ppm) 3.23 (t, J = 7.4 Hz, 2H, N-CH_2_), 3.79 (t, J = 7.4 Hz, 2H, N-CH_2_), 3.83 (s, 6H, 2 × CH_3_), 4.76 (s, 4H, thiazolinyl), 7.07 (d, J = 8.7 Hz, 4H, aromatic), 7.50 (d, J = 8.7 Hz, 4H, aromatic), 7.67 (s, 2H, 2 × benzylidene). ^13^C-NMR (125 MHz, DMSO-D_6_) δ (ppm) 35.2, 49.4, 55.2, 60.2, 114.3, 126.9, 130.3, 132.4, 135.9, 160.2, 161.9, 185.3. MS: m/z for C_24_H_24_N_2_O_3_S [M + H]^+^ calculated: 420.1508; found: 421.157.

#### 4-[(*3’E,5’E*)−3’,5’-bis(p-methoxybenzylidene)-piperid-4’-one-1’-yl]−4-oxobutane-1-carboxylic acid (5)

Compound **1** (2 mmol) was dissolved in 25 mL dry chloroform containing succinic anhydride (2 mmol) and triethylamine (0.40 mL). This solution was stirred overnight at room temperature. After the addition of 10 ml of water, the water phase was extracted at pH = 4 with a further 2 × 10 ml of chloroform. Combined chloroform phase dried (Na_2_SO_4_ sicc), filtered, and evaporated. The crude product was a yellow crystalline material. Yield 75%. Mp.: 169–170 ºC after recrystallization from ethyl acetate. ^1^H-NMR (500 MHz, DMSO-D_6_) δ (ppm) 2.32 (m, 2H, N-CH_2_), 2.43 (m, 2H, N-CH_2_), 3.83 (s, 6H, 2 × CH_3_), 4.83 (m, 4H, -CH_2_-CH_2_-), 7.07 (m, 4H, aromatic), 7.53 (m, 4H, aromatic), 7.65 (s, 2H, 2 × benzylidene). ^13^C-NMR (125 MHz, DMSO-D_6_) δ (ppm) 26.9, 28.5, 42.5, 45.9, 55.2, 114.28, 114.35, 126.6, 126.9, 130.1, 130.5, 132.4, 132.6, 135.7, 160.2, 160.3, 169.8, 173.5, 185.6. MS: m/z for C_25_H_25_NO_6_ [M-H]^+^ calculated: 435.1682; found: 434.159.

#### Ethyl 2-[(*3’E,5’E*)−3’,5’-bis(p-methoxybenzylidene)-piperid-4’-one-1’-yl]−2-oxoethane-1-carboxylate (6)

Compound **1** (1 mmol) was dissolved in a mixture of anhydrous toluene (25 mL) and triethylamine (4 mmol) under gentle heating. Ethyl oxalyl chloride (1 mmol) was subsequently added while stirring, and the heating was simultaneously discontinued. Stirring was maintained until the starting material was no longer detectable on the TLC plate. After the reaction was complete, the mixture was diluted with water (20 mL). The organic phase was subsequently extracted with chloroform (3 × 15 mL). The combined organic layers were dried (Na_2_SO_4_ sicc) and evaporated, and the crude yellow product was recrystallized from methanol. Yield 70%. Mp.: 145–146 ºC. ^1^H-NMR (500 MHz, DMSO-D_6_) δ (ppm) 0.89 (s, 3H, CH_3_CH_2_-), 3.83 (m, 6H, 2 × CH_3_), 3.92 (m, 2H, CH_3_CH_2_-), 4.74 (s, 2H, N-CH_2_), 4.88 (s, 2H, N-CH_2_), 7.07 (m, 4H, aromatic), 7.47 (d, J = 7.5 Hz, 2H, aromatic), 7.56 (d, J = 7.5 Hz, 2H, aromatic), 7.73 (m, 2H, 2 × benzylidene). ^13^C-NMR (125 MHz, DMSO-D_6_) δ (ppm) 13.1, 41.9, 46.0, 55.3, 61.8, 114.3, 114.4, 126.3, 126.5, 128.7, 129.1, 132.3, 132.6, 136.5, 137.2, 159.7, 160.5, 162.0, 184.3. MS: m/z for C_25_H_25_NO_6_ [M + H]^+^ calculated: 435.1682; found: 436.173.

#### 2-[(*3’E,5’E*)−3’,5’-bis(p-methoxybenzylidene)-piperid-4’-one-1’-yl]−2-oxobutane-1-carboxylic acid (7)

Compound **6** was dissolved in a solution consisting of 1 mL of water and 15 mL of methanol, and the mixture was stirred overnight. Upon completion of the reaction, as confirmed by TLC, the mixture was acidified to pH 4 and subsequently diluted with 30 mL of water. The resulting crude yellow solid was filtered and recrystallized from methanol. Yield 90%. Mp.: 158–159 ºC. ^1^H-NMR (500 MHz, DMSO-D_6_) δ (ppm) 3.84 (m, 6H, 2 × CH_3_), 4.80 (s, 2H, N-CH_2_), 4.86 (s, 2H, N-CH_2_), 7.06 (d, J = 8.7 Hz, 2H, aromatic), 7.09 (d, J = 8.7 Hz, 2H, aromatic), 7.48 (d, J = 8.7 Hz, 2H, aromatic), 7.55 (d, J = 8.7 Hz, 2H, aromatic), 7.72 (m, 2H, 2 × benzylidene). ^13^C-NMR (125 MHz, DMSO-D_6_) δ (ppm) 41.4, 46.2, 55.23, 55.24, 114.31, 114.35, 126.4, 126.6, 129.04, 129.11, 132.3, 132.5, 136.4, 136.8, 160.3, 160.4, 161.2, 163.9, 184.4. MS: m/z for C_23_H_21_NO_6_ [M-H]^+^ calculated: 407.1369; found: 406.128.

#### Ethyl 2-[(*2’E,6’E*)−2’,6’-bis(o-chlorobenzylidene)-cyclohexan-1’-one-4’-yloxy]−2-oxoethane-1-carboxylate (8)

Compound **2** was transformed to derivative **8** in the presence of ethyl oxalyl chloride and triethylamine according to the procedure described above for compound **6**. Yield 70%. Mp.: 111–112 ºC. ^1^H-NMR (500 MHz, DMSO-D_6_) δ (ppm) 1.20 (t, J = 7.1 Hz, 3H, CH_3_CH_2_-), 3.09 (m, 2H, CH_2_-CH), 3.19 (m, 2H, CH_2_-CH), 4.19 (q, J = 7.1 Hz, 2H, CH_3_CH_2_-), 5.22 (m, 1H, cyclic CH), 7.44 (m, 4H, aromatic), 7.51 (m, 2H, aromatic), 7.58 (m, 2H, aromatic), 7.84 (s, 2H, 2 × benzylidene). ^13^C-NMR (125 MHz, DMSO-D_6_) δ (ppm) 13.5, 31.4, 62.5, 70.5, 127.1, 129.6, 130.5, 130.6, 132.8, 133.4, 133.7, 135.0, 156.3, 156.8, 186.6. MS: m/z for C_24_H_20_Cl_2_O_5_ [M + H]^+^ calculated: 458.0688; found: 459.07.

#### 1-Bis[(*3’E,5’E*)−3’,5’-bis(o-fluorobenzylidene)−4’-piperidon-1’-yl]-methane-1-thione (9)

Compound **3** (2 mmol) and thiophosgene (1 mmol) were stirred in 15 mL dry toluene at room temperature in the presence of triethylamine. After one hour, 50 mL of water was added to this mixture. The separated yellow crystalline powder was filtered and recrystallized from methanol. Yield 90%. Mp.: 178–179 ºC. ^1^H-NMR (500 MHz, DMSO-D_6_) δ (ppm) 4.65 (s, 8H, 4 × N-CH_2_), 7.27–7.39 (br m, 12H, aromatic), 7.52 (m, 4H, aromatic), 7.58 (s, 4H, 4 × benzylidene). ^13^C-NMR (125 MHz, DMSO-D_6_) δ (ppm) 52.3, 116.4 (d, 2 J(C,F) = 21.6 Hz), 122.3 (d, 2 J(C,F) = 13.0 Hz), 125.2 (d, 3 J(C,F) = 3.2 Hz), 129.3 (d, 3 J(C,F) = 4.1 Hz), 131.3, 132.5 (d, 3 J(C,F) = 8.7 Hz), 133.8, 160.8 (d, 1 J(C,F) = 249.6 Hz), 184.9, 193.9. MS: m/z for C_39_H_28_F_4_N_2_O_2_S [M + H]^+^ calculated: 664.1808; found: 665.186.

### Cell cultures

All cell lines were purchased from ATCC (HEC-1A ATCC HTB-112 2024, HeLa ATCC CCL2 2023, T24 ATCC HTB-4 2022, and COS-1 ATCC CRL-1650 2024). HEC-1A human endometrium adenocarcinoma and T24 human bladder carcinoma cells were maintained in McCoys 5 A medium with Ishikawa and Grace modifications (Corning Inc., Corning, NY, USA), 10% fetal bovine serum (FBS; Capricorn Scientific GmbH, Ebsdorfergrund, Germany), and 1% penicillin/streptomycin (P/S; Capricorn Scientific GmbH, Ebsdorfergrund, Germany). HeLa human cervical carcinoma and COS-1 green monkey kidney fibroblast-like cell lines were maintained in Dulbeccos Modified Eagle Medium (DMEM), 10% FBS, and 1% P/S. The cells were maintained in a humidified atmosphere containing 5% CO_2_ at 37 °C.

### IC50 calculation

The viability of carcinoma cells was determined using a resazurin-based TOX8 cell viability assay (Merck KGaA, Darmstadt, Germany). Measurements were performed in 96-well plates using 10^4^ cells/well. The cells were treated with synthetic cyclic C_5_-curcuminoids at concentrations of 50 nM-1 µM and 1 µM-20 µM. Synthetic cyclic C_5_-curcuminoids were dissolved in DMSO. The stock solutions were further diluted in a complete cell culture medium before being added to the cells for treatment. The cells were treated for 24 h and incubated with the TOX8 reagent for 2 h. The absorbance was determined in accordance with the manufacturer’s protocol using a MultiSkan GO spectrophotometer (Thermo Fisher Scientific Inc., Waltham, MA, USA). The viability of the cells was relative to that of DMSO-treated control cells, which were designated as having 100% viability [31]. The IC50 values of the synthetic cyclic C_5_-curcuminoids were computed using GraphPad Prism 8 software (GraphPad Software, San Diego, CA, USA).

### Real-time PCR

For the experiments, 5 × 10^5^ cells/well were treated with cyclic C_5_-curcuminoids for 24 h. After collecting the cells, total RNA was isolated using an Aurum Total RNA Mini Kit (Bio-Rad Inc., Hercules, CA, USA), and the concentrations were determined by photometric measurements. Total RNA (200 ng) was used for cDNA synthesis using the iScript cDNA Synthesis Kit (Bio-Rad Inc., Hercules, CA, USA). mRNA expression analysis was performed using the CFX96 Opus Real-Time PCR System (Bio-Rad Inc., Hercules, CA, USA) with the SYBR Green protocol. For the calculation of the relative mRNA expression, the Bio-Rad CFX Maestro 2.3. Software (Bio-Rad Inc., Hercules, CA, USA) and the Livak (∆∆Ct) method were applied. The gene used for normalization was glyceraldehyde 3-phosphate dehydrogenase (GAPDH). The target gene expression levels were compared with those of the control cells, which were regarded as 1 [42]. The real-time PCR primers used are listed in Table 1.

**Table 1:** Real-time primer list

Primer	Sequence 5 → 3
long-chain-fatty-acid—CoA ligase 4 forward	TCTTGCTTTACCTATGGCTG
long-chain-fatty-acid—CoA ligase 4 reverse	CAGTACAGTCTCCTTTGCTT
arachidonate 15-lipoxygenase forward	GAGGAGGAGTATTTTTCGGG
arachidonate 15-lipoxygenase reverse	AATTTCCTTATCCAGGGCAG
arachidonate 5-lipoxygenase forward	CGCGGTGGATTCATACG
arachidonate 5-lipoxygenase reverse	GTCTTCAGCGTGATGTAC T
ferroportin forward	TTCCTTCTCTACCTTGGTCA
ferroportin reverse	AAAGGAGGCTGTTTCCATAG
ferritin heavy chain forward	GAGGTGGCCGAATCTTCCTTC
ferritin heavy chain reverse	TCAGTGGCCAGTTTGTGCAG
glyceraldehyde 3-phosphate dehydrogenase forward	TGTTCCAATATGATTCCACCC
glyceraldehyde 3-phosphate dehydrogenase reverse	CCACTTGATTTTGGAGGGAT
heme oxygenase-1 forward	ACCCATGACACCAAGGACCA
heme oxygenase-1 reverse	ATGCCTGCATTCACATGGCA
nuclear receptor coactivator 4 forward	AGTTGACCACTTTTGCTCTA
nuclear receptor coactivator 4 reverse	AGAACTCCACCAATAGCAAG
transferrin receptor 1 forward	CATGTGGAGATGAAACTTGC
transferrin receptor 1 reverse	TCCCATAGCAGATACTTCCA

### Western blot

For the Western blot experiments, 10^6^ cells/dish were used for each cell line. Cells were treated with curcuminoids for 24 h, collected, and lysed in an ice-cold lysis buffer containing 0.5% Triton-X-100. To prevent protein degradation, Complete Mini protease inhibitor cocktail (Roche Ltd., Basel, Switzerland) was mixed with the samples. Cell lysis was facilitated by sonication on ice for 2 min. Proteins were separated based on their concentrations in the samples, which were measured using a DC Protein Assay Kit (Bio-Rad Laboratories, Hercules, CA, USA). For separation, a Mini Protean Tetra Cell System (Bio-Rad Laboratories, Hercules, CA, USA) was used, and for blotting the proteins onto a nitrocellulose membrane, the Trans-Blot Turbo Transfer System and Trans-Blot Turbo RTA Transfer Kit (Bio-Rad Laboratories, Hercules, CA, USA) were used. The nitrocellulose membranes were blocked with 20 mL EveryBlot Blocking Buffer (Bio-Rad Laboratories, Hercules, CA, USA) at room temperature for 5 min. The following primary antibodies were used for western blotting according to the manufacturers protocols: anti-ferritin heavy chain (FTH) IgG (1:1000, overnight, 4 °C, Cell Signaling Technology Europe, Leiden, The Netherlands) and anti-ferroportin (FP) IgG (1:1000, 1 h, room temperature, Thermo Fisher Scientific Inc., Waltham, MA, USA). Goat anti-rabbit IgG HRP-conjugated IgG was used as the secondary antibody (1:3000; Merck Life Science Kft., Budapest, Hungary) and incubated for 1 h at room temperature. Anti-GAPDH IgG (1:3000; Merck Life Science Kft., Budapest, Hungary) served as the loading control. The development process was conducted utilizing the WesternBright ECL chemiluminescent substrate (Advansta Inc., San Jose, CA, USA). Visualization and analysis were performed using the UVItec Alliance Q9 Advanced Imaging System (UVItec Cambridge Ltd., Cambridge, UK). The target protein levels were expressed as a percentage of the target protein/loading control ratio.

### Total iron content determination

The intracellular iron content was determined using the protocol described by Riemer et al. [43]. For this assay, 5 × 10^5^ cells were cultured in 6-well plates. Cell lysis was carried out in 200 µL of 50 mM NaOH at room temperature for 2 h. Iron was liberated from the proteins using 100 µL of iron-releasing reagent for 2 h at 60 °C. To prepare the ferrozine-iron complexes, 30 µL of iron detection reagent was mixed with the samples for 30 min. The absorbance was measured at 550 nm using a Multiskan GO spectrophotometer (Thermo Fisher Scientific Inc., Waltham, MA, USA). A standard curve of FeCl_3_ in a two-fold dilution series (1–0 mM) was used to calculate the iron content. For normalization, the protein concentration of each sample was determined using a DC Protein Assay Kit (Bio-Rad Inc., Hercules, CA, USA). The iron content was determined to be µM iron/mg protein.

### Reactive oxygen species (ROS) determination

Carcinoma and control fibroblast-like cells were cultured in 96-well plates using 10^4^ cells/well. After treatment, ROS production in the cells was determined using the Fluorometric Intracellular ROS Kit (Merck Life Technologies Kft, Budapest, Hungary) according to the manufacturers instructions. The incubation period was 30 min for each of the measurements. The fluorescence intensity was measured at a λex 640/λem 675 nm wavelength using an EnSpire Multimode microplate reader (PerkinElmer, Rodgau, Germany). ROS levels were correlated with DMSO-treated control cells from each cell line and are indicated as percentages. The control ROS production was considered to be 100%.

### Thiol concentration determination

The total thiol concentrations in the different carcinoma and control fibroblast-like cells were determined using a Fluorometric Thiol Assay Kit (Merck Life Science Kft., Budapest, Hungary). Briefly, 10^6^ cells/25 cm^2^ flasks (Biologix Europe, Hallbergmoos, Germany) were treated with curcuminoids for 24 h. The cells were harvested and lysed in accordance with the manufacturers protocol. Cell pellets were lysed in 100 µL of Assay Buffer. Measurements were conducted in 96-well plates, utilizing 50 µL of both the standards and samples. The fluorescence intensity was detected at λ_Ex_ 490 nm/λ_Em_ 535 nm by EnSpire Multimode Plate Reader (Perkin Elmer, Waltham, MA, USA). The concentrations were determined using a standard curve of glutathione (GSH) and expressed in μM.

#### Glutathione peroxidase (GPx) activity measurement

The enzyme activity was measured using a Glutathione Peroxidase Assay Kit (Merck Life Science Kft., Budapest, Hungary), in accordance with the manufacturer’s protocol. Briefly, 10^6^ cells/25 cm^2^ flasks (Biologix Europe, Hallbergmoos, Germany) were treated with curcuminoids for 24 h. After lysis and centrifugation for 10 min at 14.000 × g, GPx activity was determined from 10 µL of supernatant from each sample. The optical density (OD) was measured at 340 nm using a Multiskan GO spectrophotometer (Thermo Fisher Scientific Inc., Waltham, MA, USA), and the activity values were expressed in U/L.

#### Cytochrome c enzyme-linked immunosorbent assay (ELISA) measurement

The Human Cytochrome c ELISA Kit (Thermo Fisher Scientific Inc., Waltham, MA, USA) was used to measure cytochrome c levels in accordance with the manufacturers protocol. Briefly, we used 10^6^ cells/25 cm^2^ flask (Biologix Europe, Hallbergmoos, Germany) for treatmentt with curcuminoids for 24 h. After harvesting, cell pellets were washed once with ice-cold 1 × PBS (Capricorn Scientific GmbH, Ebsdorfergrund, Germany). Then, the cells were lysed in the lysis buffer provided by the kit. The supernatants were diluted and 100 µL of each supernatant was used for ELISA. The cytochrome c concentration was determined using SkanIt software (Thermo Fisher Scientific Inc., Waltham, MA, USA). The results were expressed in ng/mL.

#### Caspase-3 assay

The caspase-3 enzyme activity was measured using a Caspase-3 Assay Kit (Merck Life Science Kft., Budapest, Hungary). The kit was used according to the manufacturers instructions. For the measurements, 10^6^ cells/flask (Biologix Europe, Hallbergmoos, Germany) were treated in 25 cm^2^ flasks. The cells were then harvested by centrifugation, washed once with 1 × phosphate buffer saline (PBS), and lysed in 300 µL of lysis buffer (50 mM HEPES, pH 7.2, 100 mM NaCl, 0.5% (v/v) Triton X-100) with shaking for 30 min on ice. After eliminating debris by centrifugation, measurements were carried out using 50 µL of the supernatant. The supernatants were incubated with the working solution for 1 h at 37 °C in the dark. The fluorescent intensity (FI) was determined at λ_Ex_ 400 nm/λ_Em_ 490 nm using an EnSpire Multimode Microplate Reader (PerkinElmer, Rodgau, Germany). FI was normalized to the absolute control values and expressed as relative FI.

#### Data analysis

There were three biological and three technical replicates, except for the viability tests, which had five technical replicates. Western blot is representative of three independent experiments. Statistical analysis was accomplished with the SPSS software (version 24.0; IBM Corporation, Armonk, NY, USA). Statistical significance was evaluated using the Students t-test with Bonferroni correction. Data are presented as mean ± standard deviation (SD). The cutoff value for statistical significance was < 0.05.

## Results

### Design and synthesis of novel cyclic C_5_-curcuminoid derivatives

As mentioned previously, the primary binding site of these cyclic C_5_-curcuminoids is the cross-conjugated *ß*-dienone moiety with two aromatic substituents on the two sides of the molecules (Fig. 3). These compounds act as thia-Michael acceptors in biochemical reactions with peptides bearing -SH function(s) in the biological place of action. Concurrently, another segment of the molecule, located opposite the carbonyl group within the central ring, functions as a "fixing tail" (secondary binding tool, Fig. 3) to facilitate enhanced binding to the targeted peptides. We assumed that binding the molecules to their biological place of action is a mutual result of primary and secondary interactions. The primary thia-Michael interaction is essential in all cancer types, whereas secondary binding can be unique to specific cancer types.

**Scheme 1:**
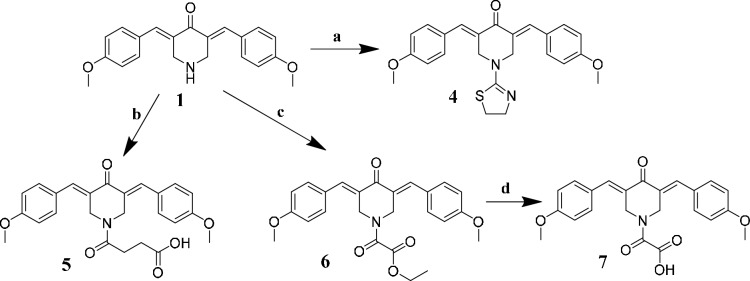
Synthesis of compounds **4**–**7** starting from **1** and reaction conditions: a = *ß*-chloroethyl isothiocyanate in dry chloroform, 1 h; b = succinic anhydride in dry chloroform, TEA, 2 h; c = ethyl oxalyl chloride in dry chloroform, TEA, 2 h; d = hydrolysis in MeOH/KOH

As a continuation of our earlier research, we aimed to further investigate the impact of the structure of the secondary binding part on the antitumor activity of our cyclic C_5_-curcuminoids. Therefore, we decided to synthesize new substituents on the central ring (Scheme 1). The starting compound (**1**) was the same in all cases to maintain a constant aromatic substituent. Compound **4** was prepared according to our previous experience with *ß*-chloroethyl isothiocyanate for the synthesis of thiazolo and/or thiazino heterocycles [44, 45]. The formation of the distinctive 2-thiazolin-2-yl substituent on the piperidone nitrogen occurs in two steps. The first is acylation with ß-chloroethyl isothiocyanate, followed by ring closure facilitated by triethylamine (TEA). Such a pharmacophore substituent has not been previously investigated in relation to curcuminoids.

Compounds **C** and **E** (Fig. 3), which have a carboxylic function in the central ring, showed very high cytotoxic activity. Therefore, we synthesized analog **5** and homologues **6** and **7** for testing on cancer cells.

Derivative **8**, analogous to compound **6** (Scheme 1), was synthesized from compound **2** through a simple acylation reaction with ethyl oxalyl chloride.

**Scheme 2:**
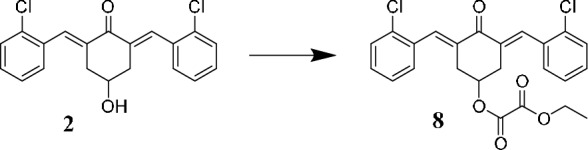
Synthesis of compound **8** starting from **2** and reaction conditions: ethyl oxalyl chloride in dry chloroform, TEA, 2 h

**Scheme 3:**
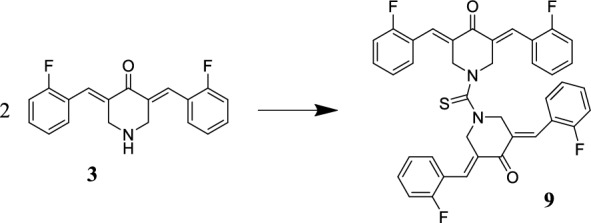
Synthesis of compound **9** from **3** and reaction conditions: two equivalents of compound **3** react with one equivalent of thiophosgene in this dimerization

The sulfur-containing substituent on compound **4** (Scheme 2) and the piperidone nitrogen form a thiocarbamide functional group. This function led us to plan the synthesis of the thiocarbamide-type dimeric compound **9,** which has two primary binding *ß*-dienone motifs in its structure (Scheme 3).

Two facts prompted the idea of preparing compound **9** for anticancer testing. On the one hand, starting compound **3** (**EF24**) is a highly auspicious cyclic C_5_-curcuminoid in this respect [46]. However, dimeric forms of similar curcuminoids display excellent tumor-selective cytotoxicity [26].

### Effects of the cyclic C_5_-curcuminoid derivatives on the viability of the carcinoma and control cell lines

Different carcinoma cells and the control cell line were treated with six synthetic cyclic C_5_-curcuminoids, and cisplatin was used as a positive control. Considering the IC50 values, compounds **6, 8**, and **9** were the most efficient against carcinoma cells, although compound **4** was also potent against T24 bladder carcinoma cells. Most of the examined compounds were more powerful than cisplatin and had similar effects on carcinoma cells (Table 2).

**Table 2:** IC50 values of cyclic C_5_-curcuminoids

IC50	HeLa	HEC-1A	T24	COS-1
Compound 4	20.96 (± 0.34) µM	15.44 (± 0.21) µM	3.28 (± 0.15) µM	23.31 (± 0.24) µM
Compound 5	4.79 (± 0.21) µM	8.54 (± 0.22) µM	8.47 (± 0.19) µM	1.48 (± 0.07) µM
Compound 6	2.0 (± 0.1) µM	3.24 (± 0.15) µM	3.16 (± 0.21) µM	1.58 (± 0.11) µM
Compound 7	7.33 (± 0.25) µM	6.58 (± 0.2) µM	5.64 (± 0.27) µM	1.35 (± 0.09) µM
Compound 8	4.3 (± 0.11) µM	4.46 (± 0.09) µM	4.29 (± 0.17) µM	5.01 (± 0.14) µM
Compound 9	4.14 (± 0.15) µM	1.21 (± 0.05) µM	0.96 (± 0.03) µM	1.76 (± 0.08) µM
Cisplatin	3.52 (± 24) µM	22.53 (± 0.31) µM	12.98 (± 0.16) µM	9.17 (± 0.21) µM

HeLa cervix carcinoma, HEC-1A endometrium adenocarcinoma, T24 bladder carcinoma, and COS-1 non-cancerous kidney fibroblast-like cells; IC50 values were calculated from the viability measurements. Values are the IC50 ± SD of three independent experiments (n = 3). Five technical replicates were performed for each experiment.

### Cyclic C_5_-curcuminoids increase the mRNA expression of ferroptosis marker genes

We examined the biological effects of cyclic C_5_-curcuminoids on genes related to ferroptosis and iron-mediated apoptosis. Based on the literature, the relative mRNA expression of nuclear receptor coactivator 4 (NCOA4), regulating ferritin heavy chain (FTH) degradation, iron importer transferrin receptor 1 (TfR1), iron exporter ferroportin (FP), iron storage gene FTH, heme-destroying, and antioxidant enzyme heme oxygenase 1 (HO-1) was analyzed.

**Fig. 4:**
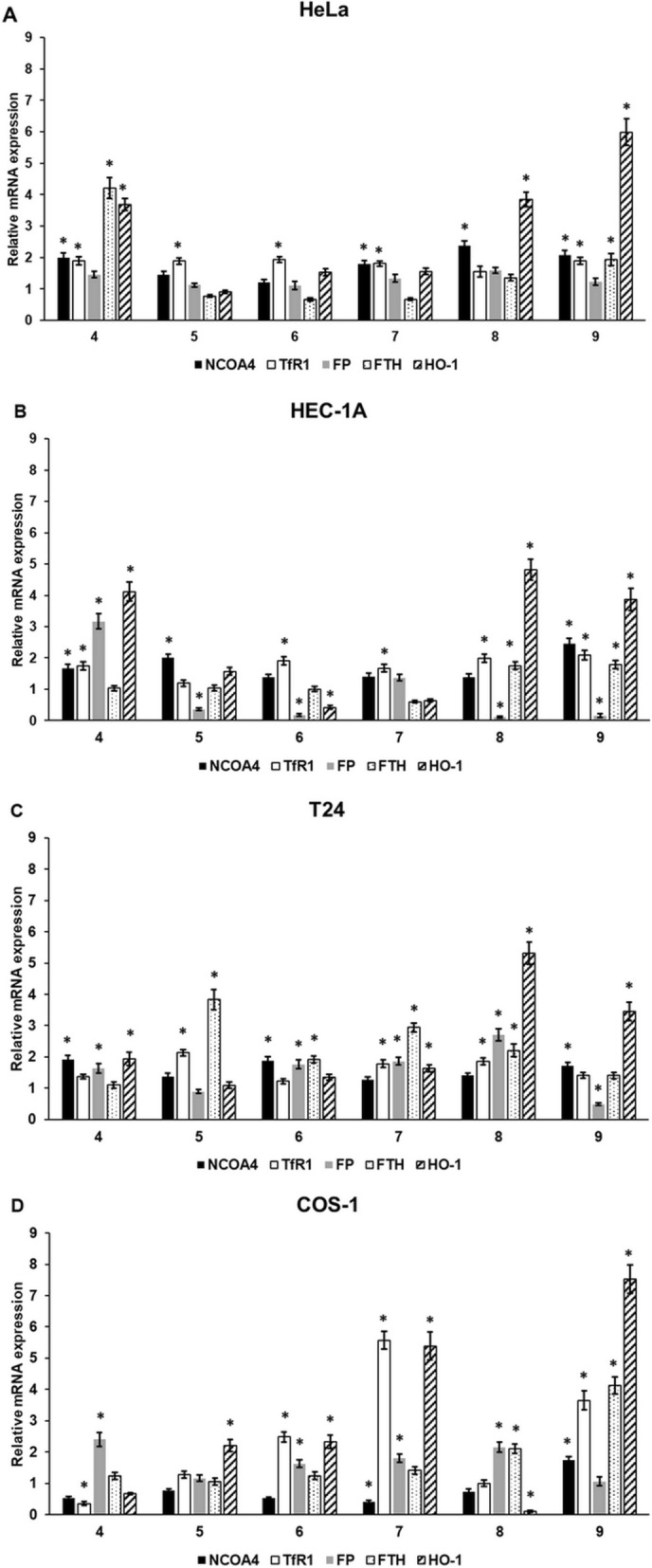
Relative mRNA expression of ferroptosis marker genes in carcinoma and control cell lines after curcuminoid treatment. The cells were treated with six different cyclic C5-curcuminoids for 24 h. After pelleting the cells, total RNA was isolated from the cells and cDNA was synthesized. Relative mRNA expression was measured using real-time PCR with the SYBR Green protocol. DMSO-treated cells were utilized as controls for gene expression analysis. The control gene expression was considered to be 1. GAPDH was used as the housekeeping gene to normalize gene expression. (**A**) Relative mRNA expression levels of NCOA4, TfR1, FP, FTH, and HO-1 in HeLa cells. (**B**) Relative mRNA expression levels of NCOA4, TfR1, FP, FTH, and HO-1 in HEC-1A cells. (**C**) Relative mRNA expression levels of NCOA4, TfR1, FP, FTH, and HO-1 in T24 cells. (**D**) Relative mRNA expression of NCOA4, TfR1, FP, FTH, and HO-1 in COS-1 control cells. Columns represent the mean ± SD of three independent experiments (n = 3). Three technical replicates were performed for each experiment in this study. Statistical significance was evaluated using the Students t-test with Bonferroni correction. The asterisk indicates p < 0.05 compared to DMSO-treated cells. Abbreviations: NCOA4-Nuclear receptor coactivator 4; TfR1-Transferrin receptor 1; FP-ferroportin; FTH-ferritin heavy chain; HO-1-heme-oxygenase-1

In HeLa cells, compounds **4, 7, 8**, and **9** significantly increased NCOA4 expression; however, only compounds **4** and **9** elevated FTH mRNA levels. TfR1 mRNA expression was increased in all cases, except for compound 8, and no significant alterations were observed in the case of FP. HO-1 levels were elevated after treatment with compounds **4, 8**, and **9** (Fig. 4A).

The elevation of NCOA4 was observed using compounds **4, 5**, and **9** in HEC-1A cells. Meanwhile, FTH levels increased after treatment with **8** and **9**. TfR1 mRNA expression increased in all cases except for treatment with compound **5**. A significant downregulation of FP expression was observed for compounds **5, 6, 7**, and **8**. HO-1 levels were elevated after treatment with compounds **4, 8**, and **9**, similar to the results observed in HEC-1A cells (Fig. 4B).

Compounds **4, 6**, and **9** significantly increased NCOA4 expression in T24 cells, whereas compounds **5, 6, 7**, and **8** elevated FTH mRNA levels. Compounds **5, 7,** and **8** increased TfR1 expression. Treatment of T24 cells with compounds **4, 6, 7**, and **8** significantly elevated the FP mRNA levels. Meanwhile, compound **9** decreased FP mRNA levels. Compounds **4, 7, 8**, and **9** increased HO-1 mRNA expression (Fig. 4C).

In COS-1 cells, NCOA4 expression was elevated only by compound **9**. FTH mRNA expression increased after the addition of compounds **8** and **9** to the culture medium. TfR1 mRNA levels were elevated after the addition of compounds **6, 7,** and **9**. A significant upregulation of FP expression was observed for compounds **4, 6, 7**, and **8**. Compounds **5, 6, 7**, and **9** also increased HO-1 mRNA expression.

Based on these results, compounds **4** and **9** were the most efficient in HeLa cells, compounds **4, 8**, and **9** were successful in HEC-1A cells, and compounds **7, 8**, and **9** were effective in T24 cells (Fig. 4D).

### Cyclic C_5_-curcuminoids increase the mRNA expression of lipid peroxidation-related genes

Next, we examined the expression of acyl-CoA synthetase long-chain family member 4 (ACSL4), arachidonate 5-lipoxygenase (ALOX5), and arachidonate 15-lipoxygenase (ALOX15), which are implicated in lipid peroxidation, a major cause of ferroptosis in carcinoma cells.

**Fig. 5:**
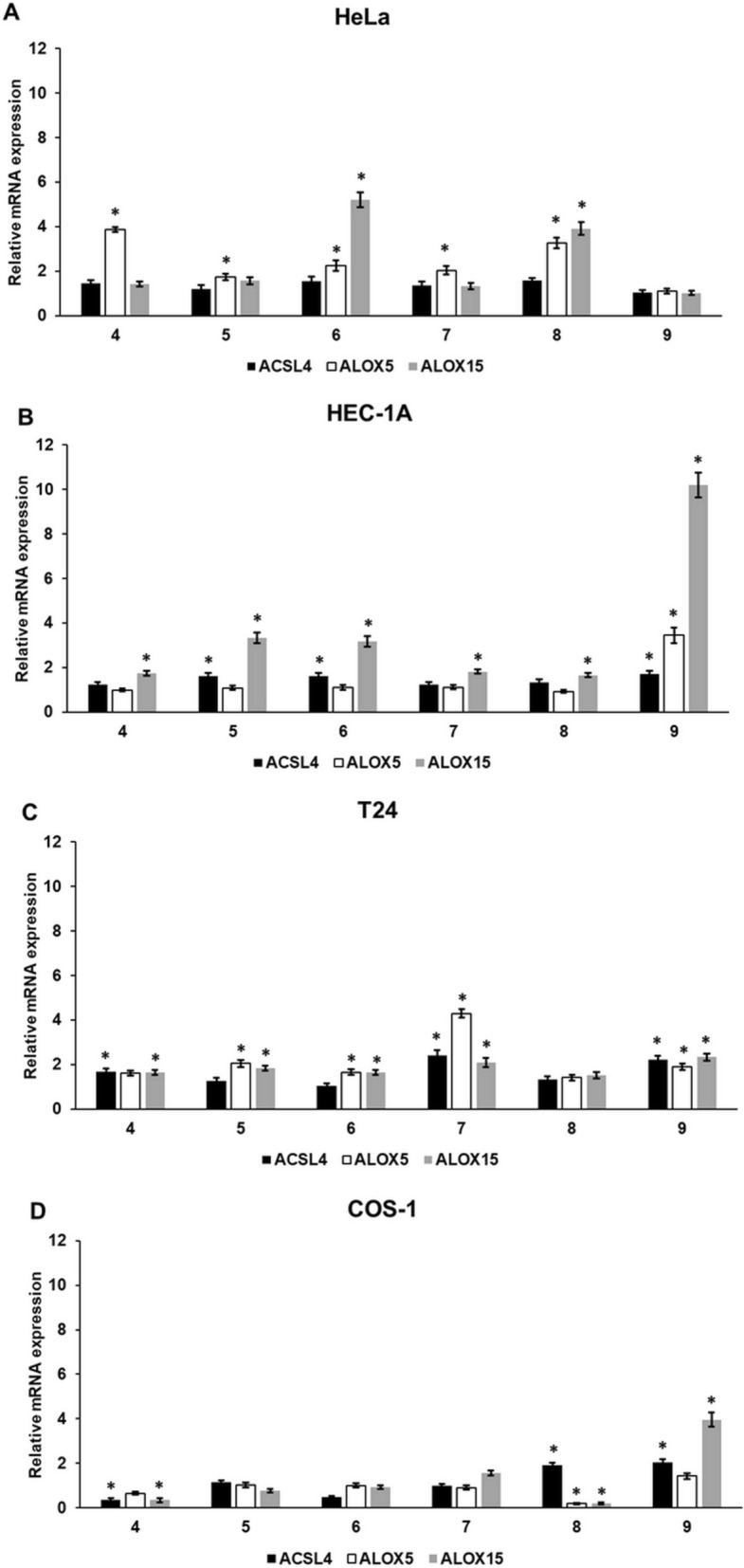
Relative mRNA expression analysis of acyl-CoA synthetase long-chain family member 4, arachidonate 5-lipoxygenase, and arachidonate 15-lipoxygenase in carcinoma and control cell lines after curcuminoid treatment. After treatments and pelleting the cells, total RNA was isolated from the cells and cDNA was synthesized. Relative mRNA expression was determined by real-time PCR using the SYBR Green protocol. DMSO-treated cells were considered as controls for gene expression analysis. The control gene expression was regarded as 1. GAPDH was used as the housekeeping gene to normalize gene expression. (**A**) Relative mRNA expression levels of ACSL4, ALOX5, and ALOX15 in HeLa cells. (**B**) Relative mRNA expression levels of ACSL4, ALO5, and ALOX15 in HEC-1A cells. (**C**) Relative mRNA expression levels of ACSL4, ALO5, and ALOX15 in T24 cells. (**D**) Relative mRNA expression of ACSL4, ALO5, and ALOX15 in COS-1 control cells. Columns represent the mean ± SD of three independent experiments (n = 3). Three technical replicates were performed for each experiment in this study. Statistical significance was evaluated using the Students t-test with Bonferroni correction. The asterisk indicates p < 0.05 compared to DMSO-treated cells. Abbreviations: ACSL4-acyl-CoA synthetase long-chain family member 4; ALOX5-arachidonate 5-lipoxygenase; ALOX15-arachidonate 15-lipoxygenase

ALOX5 expression in HeLa cells increased after the addition of all compounds, except for compound **9**. ALOX15 expression was elevated in the presence of compounds **6** and **8** (Fig. 5A).

In HEC-1A cells, ACSL4 mRNA levels increased after treatment with compounds **5, 6**, and **9**, whereas only compound **9** increased ALOX5 expression. In contrast, all compounds elevated ALOX15 mRNA expression (Fig. 5B).

In T24 cells, compounds **4, 7**, and **9** increased ACSL4 expression. Compounds **5, 6, 7**, and **9** significantly elevated ALOX5 mRNA levels, and all compounds, except compound 8, increased ALOX15 mRNA expression (Fig. 8C).

A significant downregulation, rather than augmentation, was observed in the control COS-1 fibroblast cells. Only compound **9** increased the mRNA levels of ACSL4 and ALOX15 (Fig. 5D).

Interestingly, ALOX5 expression was increased mainly in HeLa cells, whereas ALOX15 expression was elevated in HEC-1A cells, and both were increased in T24 cells.

### Cyclic C_5_-curcuminoids modify the protein levels of ferritin heavy chain and the iron exporter ferroportin

**Fig. 6:**
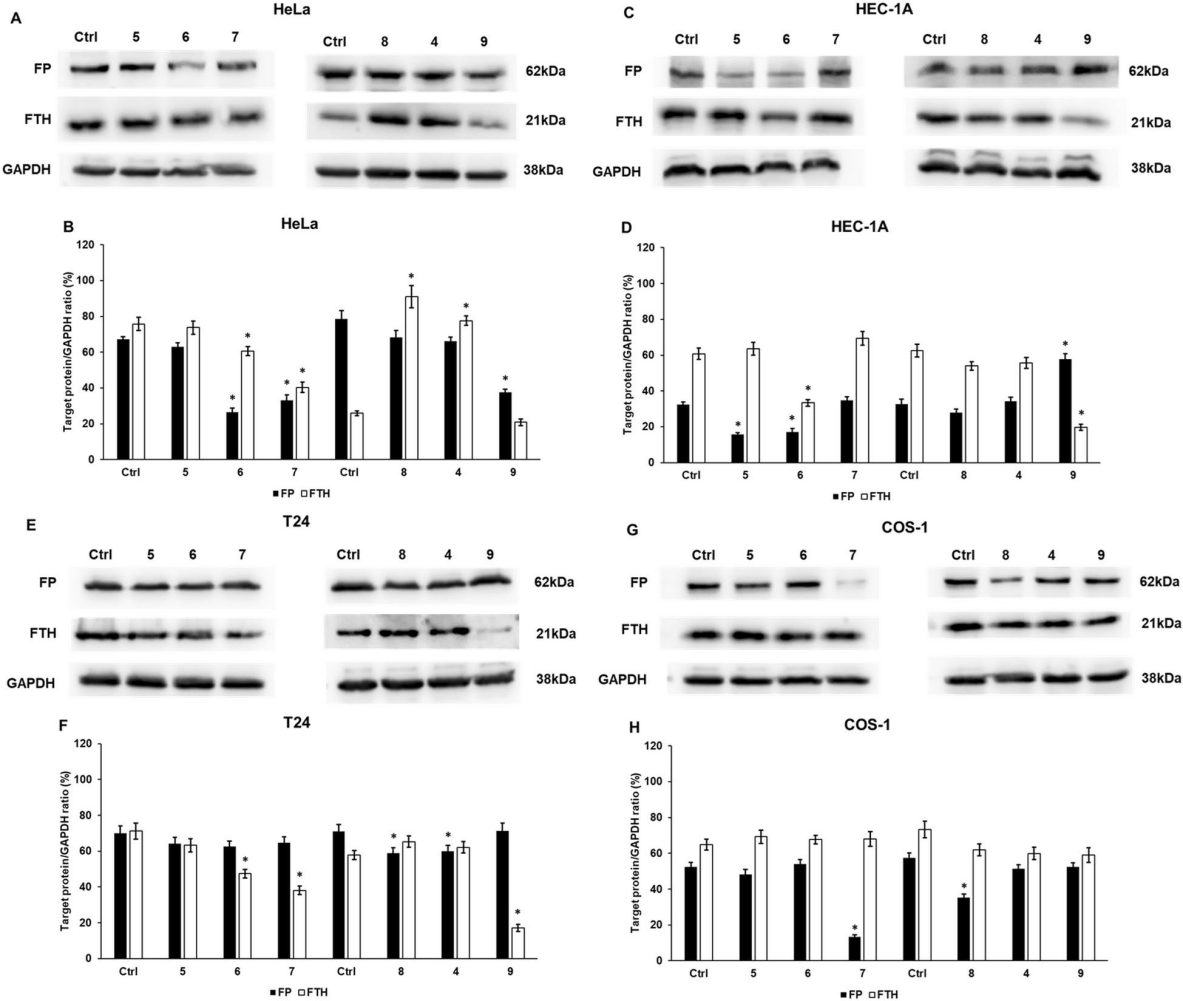
Western blot analysis of FP and FTH in carcinoma and control cell lines after curcuminoid treatment. The cells were harvested after treatment, lysed, and an equivalent quantity of protein was separated by SDS-PAGE on a 12% polyacrylamide gel. The nitrocellulose membranes were probed with anti-FP and anti-FTH antibodies. The experiments were conducted in triplicate. GAPDH served as the loading control. WBs were analyzed using the Nine Alliance software version 18.0. (**A**, **B**) Optical density analysis of FP and FTH in HeLa cells. (**C**, **D**) Optical density analysis of FP and FTH in HEC-1A cells after 24 h of treatment. (**E**, **F**) Optical density analysis of FP and FTH in T24 cells after 24 h of treatment. (**G**, **H**) Optical density analysis of FP and FTH in COS-1 cells after 24 h of treatment. Columns represent the mean ± SD of three independent experiments (n = 3). Statistical significance was evaluated using the Students t-test with Bonferroni correction. The asterisk indicates p < 0.05 compared to the DMSO control. Abbreviations: FP, ferroportin; FTH, ferritin heavy chain

Downregulation of FP, the iron exporter, and proteolysis of FTH increase the labile iron pool and trigger ferroptosis. Western blot analyses of these proteins revealed that FP and FTH protein levels decreased after treatment with compounds **6, 7**, and **9** in HeLa cells (Fig. 6A,B).

In HEC-1A cells, compounds **5** and **6** decreased FP protein levels, whereas compounds **6** and **9** significantly reduced FTH protein levels (Fig. 6C, D).

In T24 cells, protein measurements revealed a minor decrease in FP levels using compounds **6** and **7**, and significant downregulation after treatment with compounds **4** and **8**. The FTH levels decreased after treatment with compounds **6, 7**, and **9** (Fig. 6E, F).

Significant alterations in FP were observed using compounds **7** and **8** in COS-1 cells. However, the FTH levels remained close to the control levels, possibly due to the elimination of iron ions from the labile iron pool (Fig. 6G, H).

### Cyclic C_5_-curcuminoids increase ferroptosis by altering the intracellular total iron content, the ROS levels mediating oxidative stress

**Fig. 7:**
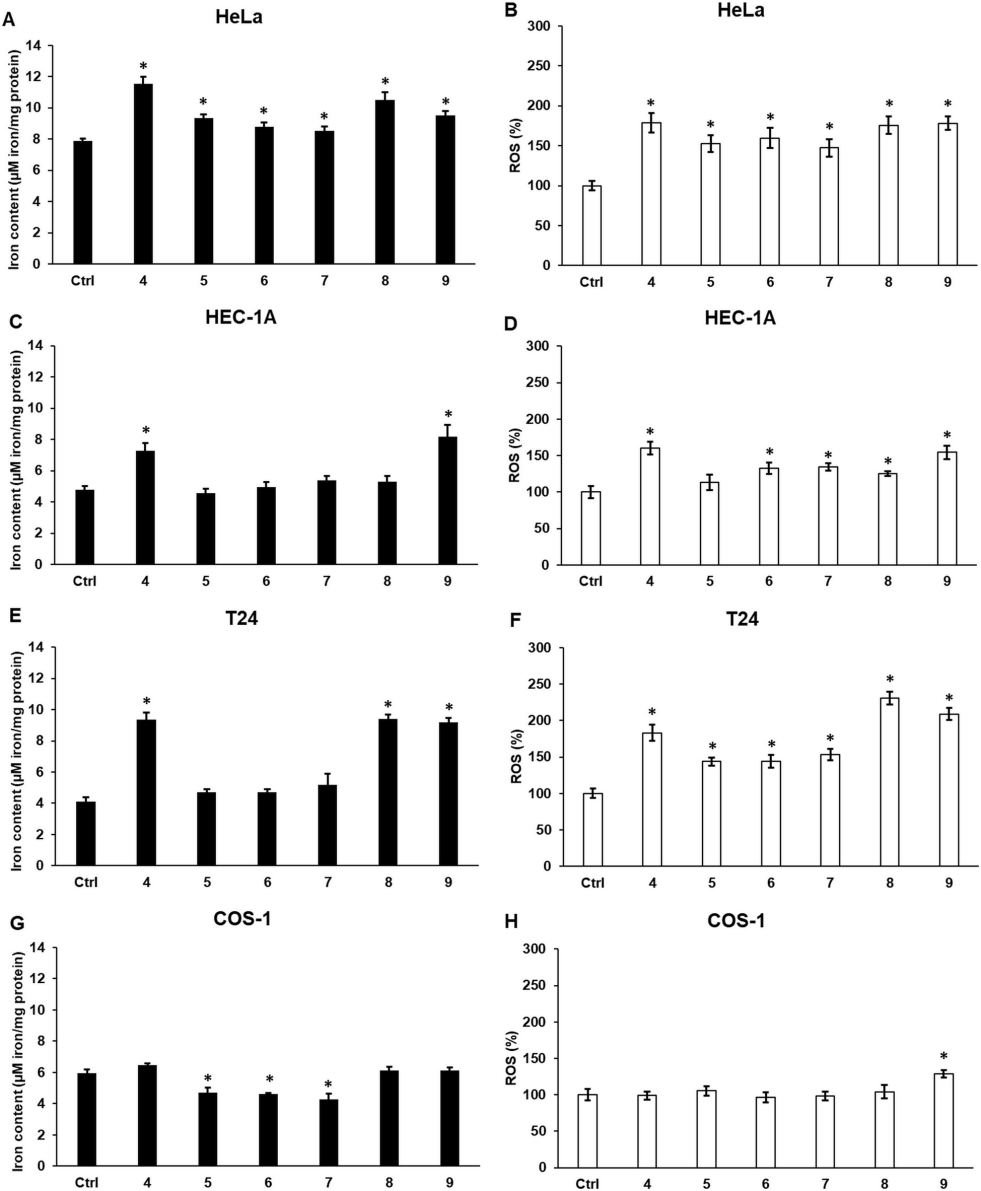
Determination of total intracellular iron content and reactive oxygen species in the carcinoma and control cell lines after curcuminoid treatment. The intracellular iron content was determined using a colorimetric ferrozine-based assay. The iron content was normalized to the intracellular protein content and expressed as µM iron/mg protein. ROS production was measured using a Fluorometric Intracellular ROS Kit. ROS levels were quantified as percentages relative to those in the control group. (**A**, **C**, **E**, **G**) Iron content of carcinomas and COS-1 control cell lines. (**B**, **D**, **F**, **H**) ROS measurements of carcinoma and COS-1 control cell lines. Columns represent the mean ± SD of three separate experiments (n = 3). Three technical replicates were performed for each experiment in this study. Statistical significance was evaluated using the Students t-test with Bonferroni correction. The asterisk indicates p < 0.05 compared to the DMSO control. Abbreviations: ROS, reactive oxygen species

Elevated intracellular iron levels, particularly in the labile iron pool, can contribute to oxidative stress via the Fenton reaction. Total intracellular iron measurements revealed that all compounds significantly elevated the iron content in HeLa cells, similar to the increase in ROS production (Fig. 7A,B).

In HEC-1A cells, compounds **4** and **9** significantly elevated iron content, whereas compounds **6, 7**, and **8** caused a slight increase. ROS production increased with increasing iron content (Fig. 7C, D).

Compounds **4, 8,** and **9** significantly augmented the total iron content, whereas the other three compounds caused minimal changes in the iron levels in T24 cells. ROS production increased according to the iron levels (Fig. 7E, F).

In the control cell line, compounds **5, 6**, and **7** significantly reduced the total iron content, whereas only compound **9** increased the ROS levels (Fig. 7G, H).

We can conclude that the examined synthetic C_5_-cyclic curcuminoids increased the total intracellular iron content and ROS production in carcinoma cells.

### Cyclic C_5_-curcuminoids change the thiol levels and decrease the glutathione peroxidase (GPx) activity of the carcinoma cells

The total thiol concentration and GPx activity of the treated carcinoma cells were determined to further examine lipid peroxidation.

**Fig. 8:**
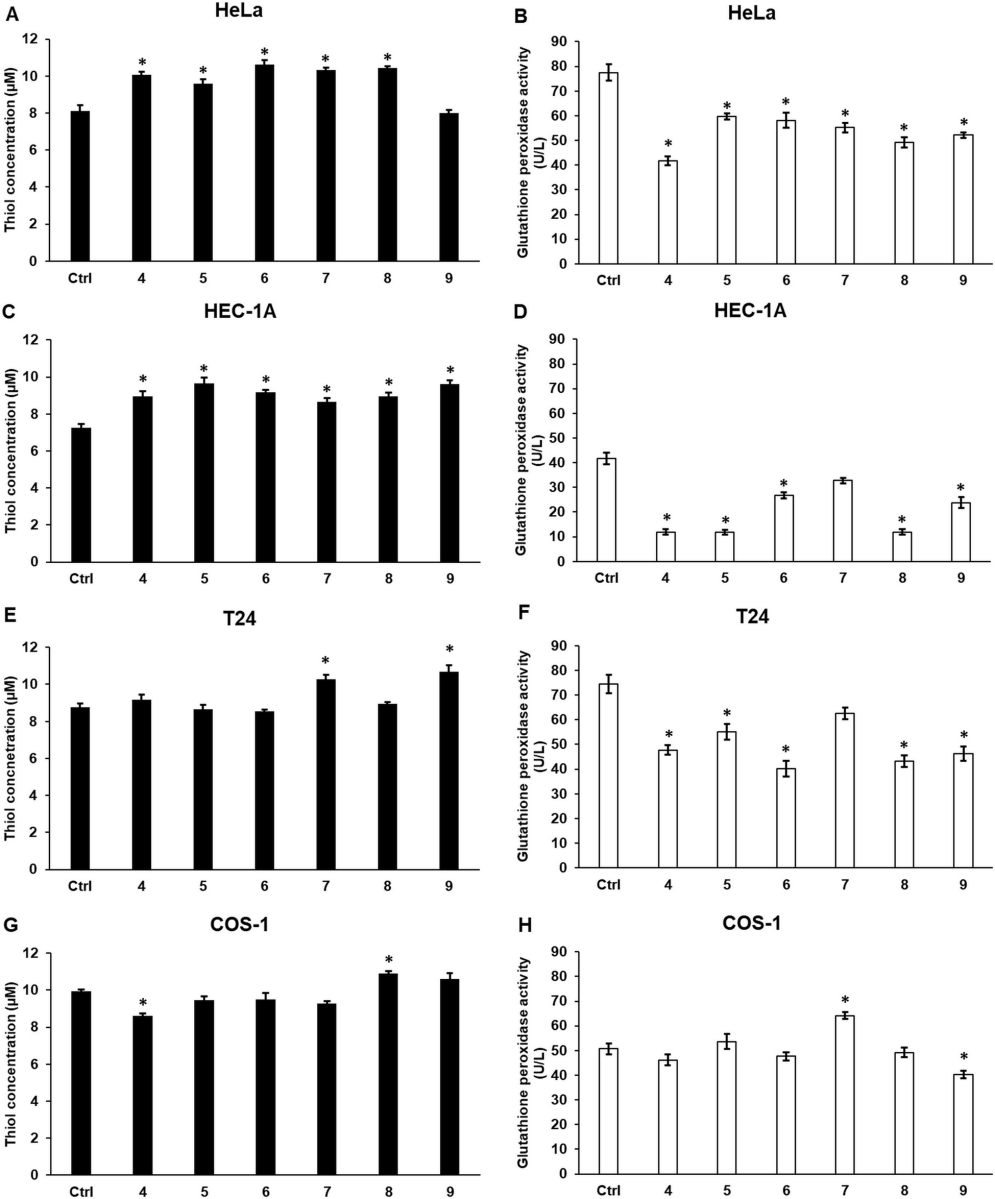
Thiol concentration and glutathione peroxidase activity measurements of carcinoma and control cell lines after curcuminoid treatment. The total thiol content of the cells was measured using the Fluorometric Thiol Assay, and GPx activity was determined using the Glutathione Peroxidase Assay Kit. (**A**, **C**, **E**, **G**) Thiol concentrations in carcinomas and COS-1 control cell lines. (**B**, **D**, **F**, **H**) GPX activity of the carcinoma and COS-1 control cell lines. Columns show the mean ± SD of three independent experiments (n = 3). Three technical replicates were performed for each experiment in this study. Statistical significance was evaluated using the Students t-test with Bonferroni correction. The asterisk indicates p < 0.05 compared to the DMSO control. Abbreviations: GPx, glutathione peroxidase

In HeLa cells, thiol levels increased, except for compound **9**, and GPx activity was reduced compared to that in DMSO-treated control cells (Fig. 8A, B).

In HEC-1A cells, all compounds elevated thiol concentrations and decreased GPx activity, except for compound **7** (Fig. 8C, D).

T24 cells showed fewer alterations in thiol levels. Only treatment with compounds **7** and **9** increased thiol levels. Similar to HEC-1A cells, all compounds lowered GPx levels, except for compound **7** (Fig. 8E, F).

In the control COS-1 cells, only compound **8** increased the thiol concentration, and compound **9** significantly decreased GPx activity (Fig. 8.G, H).

These results suggest that carcinoma cells activate protection against lipid peroxidation by elevating thiol levels, although decreased GPx activity supports reduced defensive activity.

### Cyclic C_5_-curcuminoids increase the levels of cytochrome c and elevate the activity of caspase-3, enhancing apoptosis

**Fig. 9:**
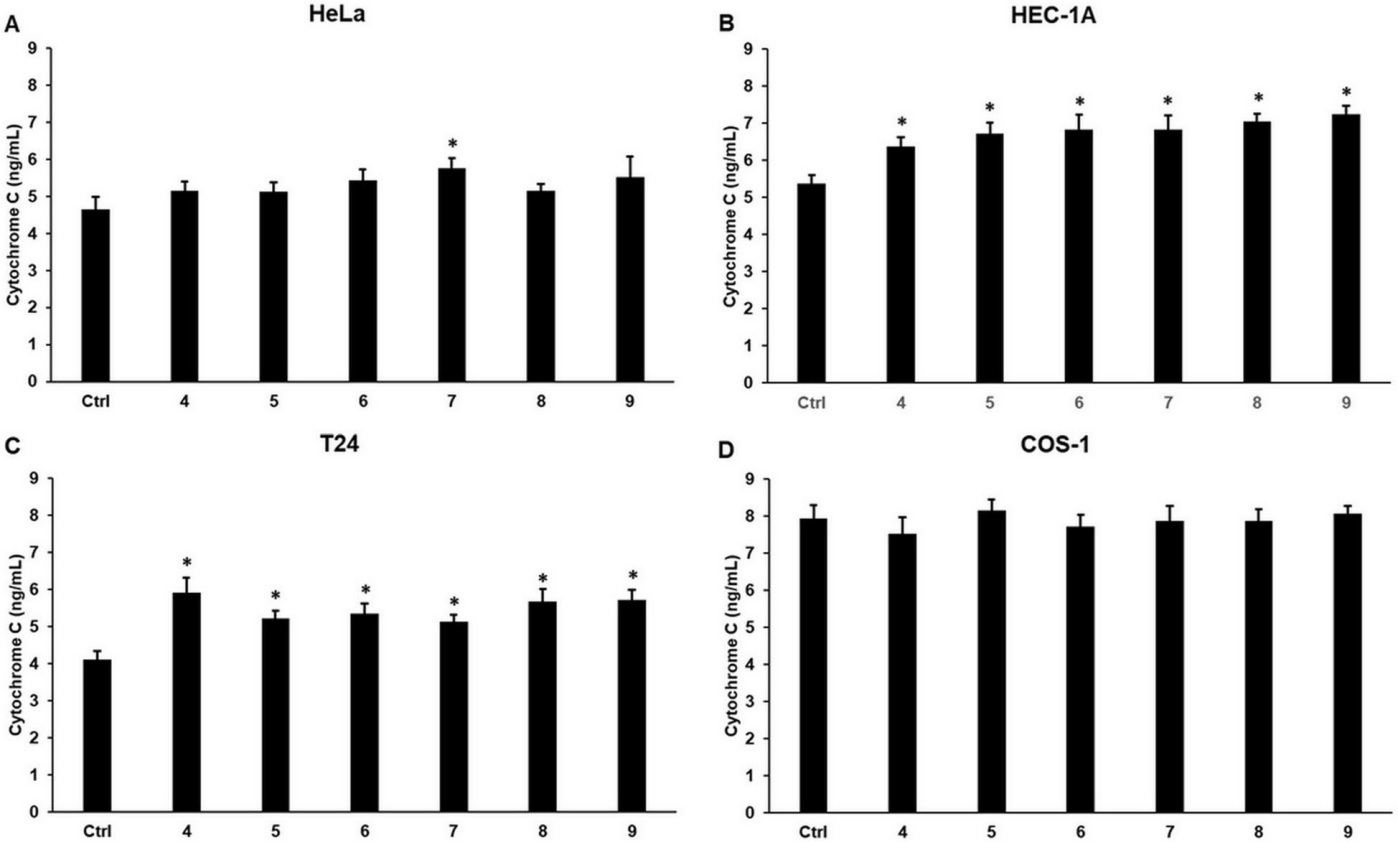
Cytochrome c concentration measurements in carcinoma and control cell lines after curcuminoid treatment. Cytochrome c levels were measured using the Human Cytochrome c ELISA Kit, in accordance with the manufacturers protocol. (**A**) Cytochrome c concentration in HeLa cells. (**B**) Cytochrome c levels in HEC-1A cells. (**C**) Cytochrome c concentration in T24 cells. (**D**) Cytochrome c content in COS-1 cells. Columns represent the mean ± SD of three separate experiments (n = 3). Three technical replicates were performed for each experiment in this study. Statistical significance was evaluated using the Students t-test with Bonferroni correction. The asterisk indicates p < 0.05 compared to the DMSO control

The release of cytochrome c from the mitochondria is a valuable indicator of the activation of apoptosis. In HeLa cells, only compound **7** increased cytosolic cytochrome c levels (Fig. 9A). In HEC-1A and T24 carcinoma cells, all compounds successfully elevated cytochrome c concentrations (Fig. 9B, C). We did not observe significant alterations in cytochrome c levels in the COS-1 cells (Fig. 9D).

**Fig. 10:**
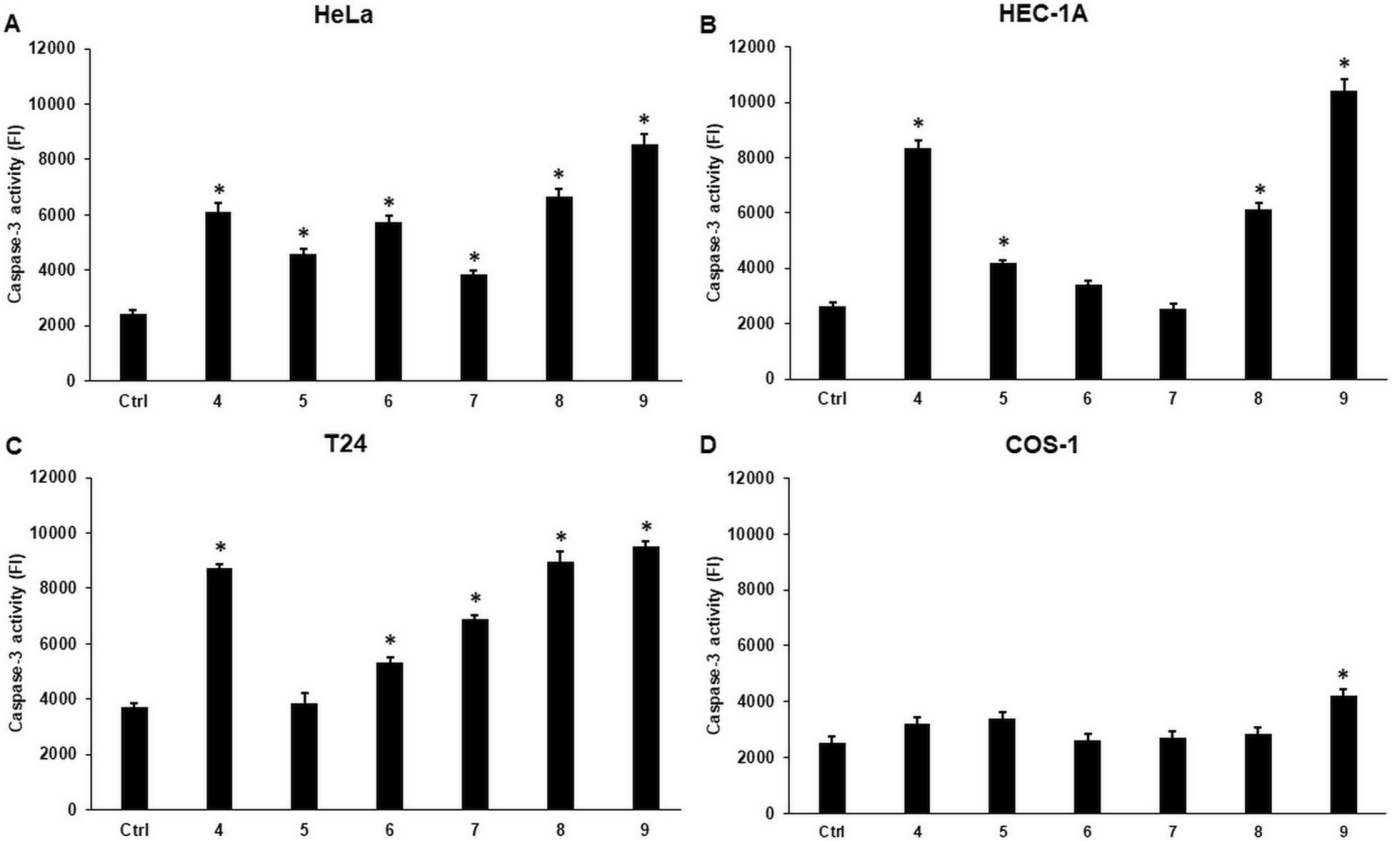
Caspase-3 activity in carcinoma and control cell lines after curcuminoid treatment. The caspase-3 activity was measured using a Caspase-3 Assay Kit in accordance with the manufacturers protocol. FI was normalized to the absolute control values and expressed as relative FI. (**A**) Caspase-3 activity in HeLa cells. (**B**) Caspase-3 activity in HEC-1A cells. (**C**) Caspase-3 activity in T24 cells. (**D**) Caspase-3 activity in COS-1 cells. Columns represent the mean ± SD of three independent experiments (n = 3). Three technical replicates were performed for each experiment in this study. Statistical significance was evaluated using the Students t-test with Bonferroni correction. The asterisk indicates p < 0.05 compared to the DMSO control

The caspase-3 activity was also measured to demonstrate the initiation of apoptosis. Although cytochrome c levels did not support pro-apoptotic events, all compounds increased caspase-3 activity in HeLa cells (Fig. 10A). In HEC-1A cells, compounds **4, 5, 8**, and **9** increased caspase-3 activity (Fig. 10B). In T24 cells, all compounds elevated caspase-3 activity, except for compound **5** (Fig. 10C). In COS-1 cells, only compound **9** raised the caspase-3 activity (Fig. 10D).

These findings suggest that alternative pro-apoptotic pathways may be involved in the activation of effector caspase-3 in HeLa cells. Meanwhile, in the case of HEC-1A and T24 cells, cytochrome c may act as an activator of apoptosis, but additional regulators could work together with cytochrome c in apoptotic processes.

## Discussion

Cancer affects one-third of the global human population. Therefore, considerable research has been conducted to develop novel treatment strategies. Cancer remains the second leading cause of mortality [47]. Research in anticancer pharmacology has resulted in the development of efficacious and targeted therapeutic strategies aimed at inhibiting tumor proliferation and metastasis [48]. Biological therapies, such as monoclonal antibodies, peptide drugs, small-molecule inhibitors, targeted protein degradation (PROTAC), and other new genetic therapies, such as mRNA therapy, CRISPR therapy, and tumor vaccines, are challenging in many respects [48–55].

Investigating natural compounds within pharmaceutical research has also assisted in the identification of novel and promising active substances [2].

C_5_-curcumin, a cyclic C_5_-curcuminoid, and synthetic cyclic C_5_-curcuminoids have been proven to be promising antitumor molecules with more favorable pharmacokinetic properties than curcumin [6, 7]. The antioxidant and anti-inflammatory effects of synthetic cyclic C_5_-curcuminoids have also been investigated in models of neurodegenerative diseases, with promising results [42, 56].

Numerous studies have examined the antitumor properties of cyclic C_5_-curcuminoids both in vitro and in vivo with promising results [57, 58]. The pleiotropic effects of cyclic C_5_-curcuminoids have been demonstrated in numerous types of malignant tumors. Synthetic C_5_-curcuminoids have been tested in glioma cell lines with high potency for decreasing proliferation [31]. In pancreatic cancer, curcumin analogs have been demonstrated to act as PI3K inhibitors, reduce Bcl2 levels, and elevate procaspase-3 protein levels. [59]. ROS-generating and pro-apoptotic effects of curcuminoids have also been reported in colorectal cancer [60, 61]. Curcumin and its derivatives have been investigated in various breast cancer cell lines, including T47D, MCF7, MDA-MB-231, MDA-MB-435, MDA-MB-468, BT20, SK-BR-3, ZR75-1, and Hs578T. These studies have demonstrated the compounds ability to inhibit cell proliferation, induce G2/M cell cycle arrest, and suppress metastasis, migration, invasion, and angiogenesis, while also activating apoptosis [62–68]. Given the extensive data available in the literature, we have opted to focus on bladder carcinoma and endometrial adenocarcinoma, where the effects of curcumin have been studied, but not those of curcumin analogs and cyclic C_5_-curcuminoids.

In this study, six new synthetic cyclic C_5_-curcuminoids were investigated in three carcinoma and adenocarcinoma cell lines: HeLa cervical carcinoma, HEC-1A endometrial adenocarcinoma, and T24 bladder carcinoma. The healthy fibroblast-like cell line COS-1 was employed as the control cell line in the experiments. The COS-1 cell line was derived from the CV-1 green monkey epithelial cells. Although its morphology resembles that of fibroblasts, it retains several epithelial cell characteristics, such as the ability to form polarized monolayers and the presence of microvilli on the cell surface. According to the literature, the COS-1 cell line is appropriate for research on epithelial processes [69] and can serve as a control for anti-tumor drug tests [67, 68, 70–72]. We focused on the signs of ferroptosis at both mRNA and protein levels in the treated cells. To confirm the initiation of ferroptosis, intracellular iron levels, ROS production, thiol concentrations, GPx activity, cytochrome c concentrations, and Caspase-3 activity were determined.

Iron is a vital element for all living organisms. Iron is implicated in the initiation, growth, proliferation, and migration of malignant cells, and in the appearance of metastasis [73]. However, cancer cells have been shown to accumulate iron for cell division and other cellular functions [74]. However, when iron levels rise in the labile iron pool, it can start the Fenton reaction. This reaction creates ROS, which can harm cells and trigger ferroptosis, causing cell death [75]. Therefore, the evolution of iron-based therapies provides new possibilities for antitumor treatment [76]. Certain anticancer drugs and curcumin derivatives also affect these pathways.

The initiation of ferroptosis can be described by elevated mRNA expression of specific genes [77]. These genes are related to iron homeostasis, particularly iron transport and storage, and lipid peroxidation. Consequential iron overload, increased ROS production, downregulation of the cystine-glutamate antiporter system, glutathione depletion, and the release of arachidonic acid mediators drive ferroptosis [78].

Synthetic cyclic C_5_-curcuminoids affect NCOA4, enhancing ferritinophagy and proteolysis of FTH, which is responsible for iron storage. Consequently, the iron level in the reactive iron-containing labile iron pool is elevated in the cells [79]. The results indicate that certain cyclic C_5_-curcuminoids were able to upregulate the expression of FTH and the iron importer TfR1. The iron exporter FP was also analyzed, and distinct results were obtained for the three cancerous cell lines. FP mRNA levels were not altered in HeLa cells. However, it was downregulated in HEC-1A cells and elevated in T24 cells, suggesting that curcuminoids have different effects on cellular iron metabolism. Compounds **4** and **9** were the most efficient in HeLa cells. Compounds **4, 8**, and **9** were effective in HEC-1A cells, while compounds **7, 8,** and **9** were effective in T24 cells. These results indicate that compound **9** can affect all three cancerous cell types and COS-1 cells.

At the protein level, the iron storage protein FTH and iron exporter FP were examined, which are suitable for predicting modifications in the labile iron pool [80]. Interestingly, compound **9** was the most efficient in decreasing FTH levels in carcinoma cells but not in COS-1 cells. More varied results were obtained for the iron release. Compound **6** decreased FP protein levels in all tumor cell types but not in COS-1 cells. However, compound **7** reduced FP expression in HeLa and T24 cells, similar to that observed in COS-1 cells. Compound **9** downregulated FTH and FP only in HeLa cells.

Although compound **9** appeared to be the most potent curcuminoid at the mRNA level, it was only effective in reducing FTH protein levels. In contrast, compound **6** exhibited a greater efficacy in reducing FP. To initiate ferroptosis, the augmentation of intracellular iron levels is a crucial step in promoting free radical production and lipid peroxidation [76]. In our experiments, all compounds significantly elevated the iron and ROS levels in HeLa cells. In HEC-1A cells, compounds **4** and **9**, and in T24 cells, compounds **4, 8**, and **9** significantly increased iron and ROS levels. Compound **9** appeared to be the most effective in triggering oxidative stress and possibly ferroptosis. In COS-1 control cells, iron content decreased after treatment, which may have attenuated the activation of ferroptosis.

Several research groups have revealed the various effects of curcuminoids on different malignant cells. At different concentrations, these active substances can affect ROS production, lipid peroxidation, intracellular ATP levels, energy metabolism, and cell death by modulating distinct signal transduction pathways [81–83]. An increase in ROS levels implies the initiation of lipid peroxidation [84]. Therefore, further measurements were conducted to monitor the expression of lipid peroxidation-related genes. Arachidonate 5-lipoxygenase (ALOX5) mRNA levels were increased in HeLa cells. Arachidonate 15-lipoxygenase (ALOX15) levels were increased in HEC-1A cells. Both ALOX genes were upregulated in T24 cells. However, the strengths of the impacts of the different compounds were quite different. Although compound **9** appeared to be the most powerful, it was ineffective in altering lipid peroxidation-related genes in HeLa cells. However, the other five compounds upregulated ALOX5 expression in cervical carcinoma cells. The effects of cyclic C_5_-curcuminoids on acyl-CoA synthetase long-chain family (ACSL4) are entirely disparate. ACSL4 mRNA expression was modified in HEC-1A by compounds **5, 6**, and **9** and in T24 cells by compounds **4, 7**, and **9**. Only minor elevations were revealed in HeLa cells treated with all compounds.

Although the thiol levels were mainly increased in cancer cells, all compounds reduced GPx activity, with the exception of compound **7**. These results suggest that cancer cells attempt to protect themselves from oxidative damage by increasing their free thiol levels. Nonetheless, decreased GPx activity may weaken the defensive capacity [85, 86].

Regular mitochondrial activity is fundamental to ensure that cells provide energy for cellular functions. Moreover, the mitochondria are sites of heme and iron-sulfur cluster synthesis, which are required for physiological activities [87]. The decline in ATP production results in energy stress, which prevents ferroptosis via the activation of AMP-activated protein kinase (AMPK) [88]. In contrast, mitochondria are implicated in the control of apoptosis. It has been revealed that curcumin and other curcuminoids activate caspase-3 and enhance apoptosis in Jurkat T cell leukemia, glioblastoma multiforme 8401 cells, and melanoma cells [89–91].

Cytochrome c is a mitochondrial protein whose release leads to the activation of effector caspase-3, resulting in increased levels of cleaved caspase-3. Cytochrome c is released from the mitochondrial membrane through distinct mechanisms. Increased mitochondrial ROS production targets cardiolipin in the mitochondrial membrane, resulting in the discharge of cytochrome c [92]. BID also activates the BAX/BAK channel in the mitochondrial membrane. This starts the permeability transition pore complex (PTPC) and makes the outer membrane of the mitochondria more permeable. As a result, cytochrome c is released [93].

Cytochrome c measurements revealed the different effects of cyclic C_5_-curcuminoids on carcinoma cells. In HEC-1A and T24 cells, all compounds significantly increased cytochrome c concentration, but were ineffective (except compound **7**) in HeLa cells and COS-1 control cells.

Interestingly, caspase-3 activity significantly increased in HeLa cells, although cytochrome c levels did not indicate pro-apoptotic events. A possible reason for these results is the involvement of an alternative apoptotic pathway. This involves the liberation of Smac/DIABLO proteins, inactivation of inhibitors of apoptosis proteins (IAPs), and release of caspase-3, which results in apoptosis [94]. In HEC-1A and T24 cells, caspase-3 activity was significantly upregulated, supporting the apoptotic effects of the examined synthetic cyclic C_5_-curcuminoids.

We examined the effects of six synthetic cyclic C_5_-curcuminoids on three cancer cell lines: HeLa, HEC-1A, and T24. However, the examined curcuminoids exerted distinct responses in HeLa cells compared to HEC-1A and T24 cells. These data support that cyclic C_5_-curcuminoids possess different mechanisms of action. Compound **4** also efficiently increased iron accumulation and ROS production and reduced antioxidant defense in all three carcinoma cell lines, indicating ferroptosis. The apoptosis-inducing activity of curcuminoids was also observed. Compound **9** successfully induced caspase-3 activity in cancer cells and triggered apoptosis in the control cell line, COS-1. Analysis of data from the COS-1 control cell line and the three cancer cell lines indicated that compound **4** is a promising synthetic cyclic C_5_-curcuminoid. However, we must consider that the IC50 values of compound **4** are 3–13-fold higher than those of compound **9**.

Thiazole and thiazine represent highly promising pharmacophores. However, the incorporation of such substituents under preparative conditions presents significant challenges, which have thus far impeded their integration. Overcoming these challenges may unlock new possibilities in the field.

We emphasize that our in vitro experiments were specifically designed to identify compounds that could be considered as potential antitumor drug candidates. Additionally, we are evaluating these synthetic compounds in brain-derived tumor cell lines to elucidate their potential applications and identify the most effective cyclic C_5_-curcuminoids.

## Conclusions

Our findings, in conjunction with the existing literature, indicate that the anticancer potential of these cyclic C_5_-curcuminoids varies across different cancer types. We propose that finding at least one effective derivative among them against different malignant tumor cells is highly possible owing to their pleiotropic characteristics. Their efficacy strongly hinges on the binding ability of the primary (substituted aromatic *ß*-dienone motif as a thia-Michael acceptor) and secondary (substituents on the central ring as an additional fixing tail) binding tools over the -SH group(s) containing peptides, which are necessary for the survival of cancer cells. These observations suggest that primary binding is fundamental for all cancer types. Secondary interactions are typical structural details of a targeted peptide at the site of action.

Based on the diverse effects of the six novel synthetic cyclic C_5_-curcuminoids on the three cancer cell types, we hypothesized that ferroptosis and/or apoptosis are enhanced in the tumor cells. However, their mechanisms of action differ and involve divergent target molecules and signal transduction pathways. We further aimed to identify a substituent that exhibited optimal activity, characterized by selectivity and favorable kinetics. To verify the therapeutic potential of the selected cyclic C_5_-curcuminoids, it is essential to perform formulation and in vivo studies utilizing cancerous animal models. Moreover, pharmacokinetic and off-target toxicity studies should be conducted in vivo. Further examination of possible targets could help elucidate which compounds are more suitable for consideration as prospective antitumor drug candidates.

## Supplementary Information

Additional file 1. 

Additional file 2

## Data Availability

The datasets used and/or analyzed during the current study are available from the corresponding author upon reasonable request.

## Abbreviations

ACSL4: Acyl-CoA synthetase long-chain family member 4
ALOX: Arachidonate lipoxygenase
AMPK: AMP-activated protein kinase
DMSO: Dimethyl sulfoxide
ELISA: Enzyme-linked immunosorbent assay
FBS: Fetal bovine serum
FI: Fluorescent intensity
FP: Ferroportin
FTH: Ferritin heavy chain
GAPDH: Glyceraldehyde 3-phosphate dehydrogenase
GPx: Glutathione peroxidase
HO-1: Heme oxygenase-1
HRP: Horse radish peroxidase
IC50: Half maximal inhibitory concentration
IgG: Immunoglobulin G
MOMP: Mitochondrial outer membrane permeabilization
NCOA4: Nuclear receptor coactivator 4
P/S: Penicillin/streptomycin
PBS: Phosphate buffer saline
PTPC: Permeability transition pore complex
ROS: Reactive oxygen species
TBST: Tris-buffered saline with Tween 20
TEA: Triethylamine
TfR1: Transferrin receptor 1
