# Supplementary material for: Novel cyclic C5-curcuminoids possess anticancer activities against HeLa cervix carcinoma, HEC-1A adenocarcinoma, and T24 bladder carcinoma cells
Source: Cancer Cell Int. 2025 Dec 3;25:431. doi: 10.1186/s12935-025-04077-2 (PMC12676898; doi:10.1186/s12935-025-04077-2)
Supplement: Supplementary file 1 — Additional file 1. [file 12935_2025_4077_MOESM1_ESM.pdf]

All ^{1}H and ^{13}C NMR spectra were in good accordance with the expected structures. The ^{1}H-NMR and ^{13}C-NMR spectroscopic data for the chemical shifts and coupling constants, together with the proton assignments of the prepared compounds **4-9** are listed in section 2.2. The presented homogeneous *E,E*-configuration of the benzylidene double bonds in the structures **4-9** is based on our earlier studies [32]. The anisotropic effect of the cyclic carbonyl group over the neighboring benzylidene protons can be the main reason for the formation of *E,E*-geometrical isomers.

Compound 4:

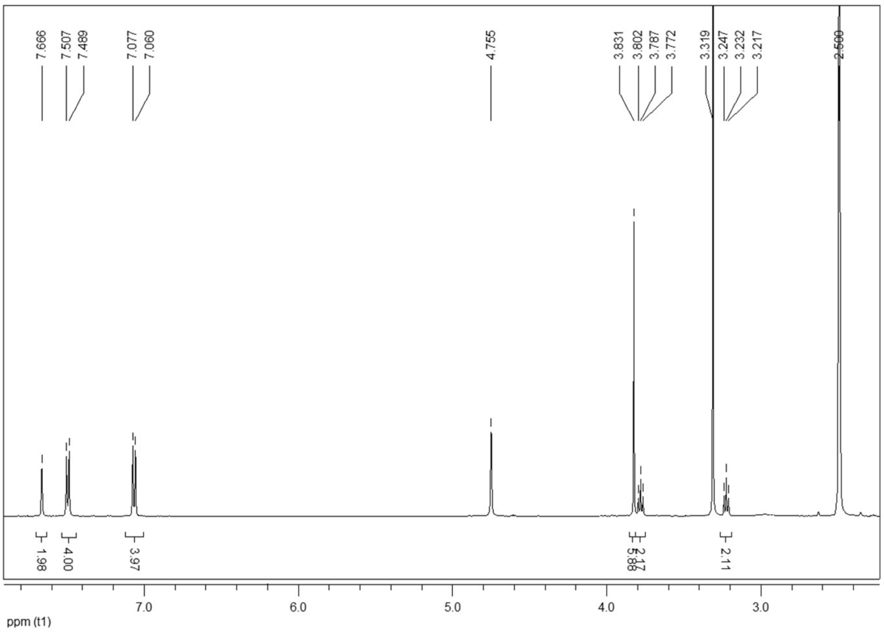

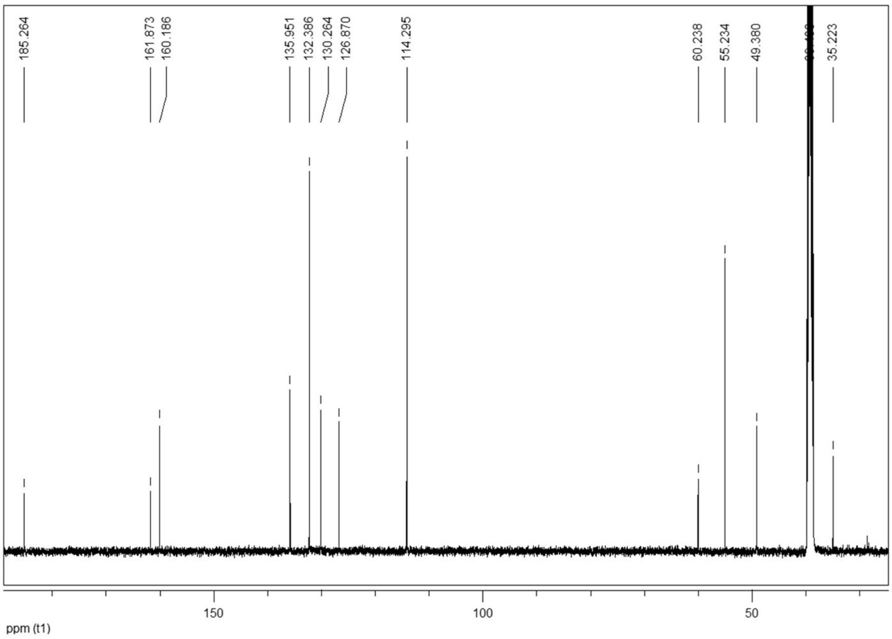

Compound 5:

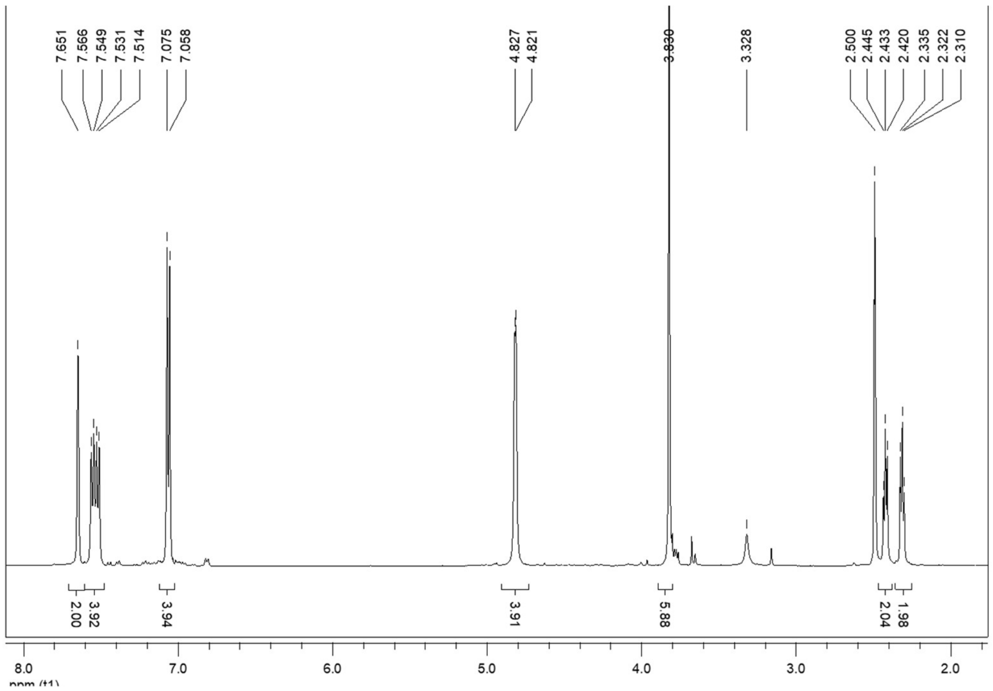
7.651
7.566
7.549
7.531
7.514
7.075
7.058
4.827
4.821
3.830
3.328
2.500
2.445
2.433
2.420
2.335
2.322
2.310
2.00
3.92
3.94
3.91
5.88
2.04
1.98
8.0
7.0
6.0
5.0
4.0
3.0
2.0

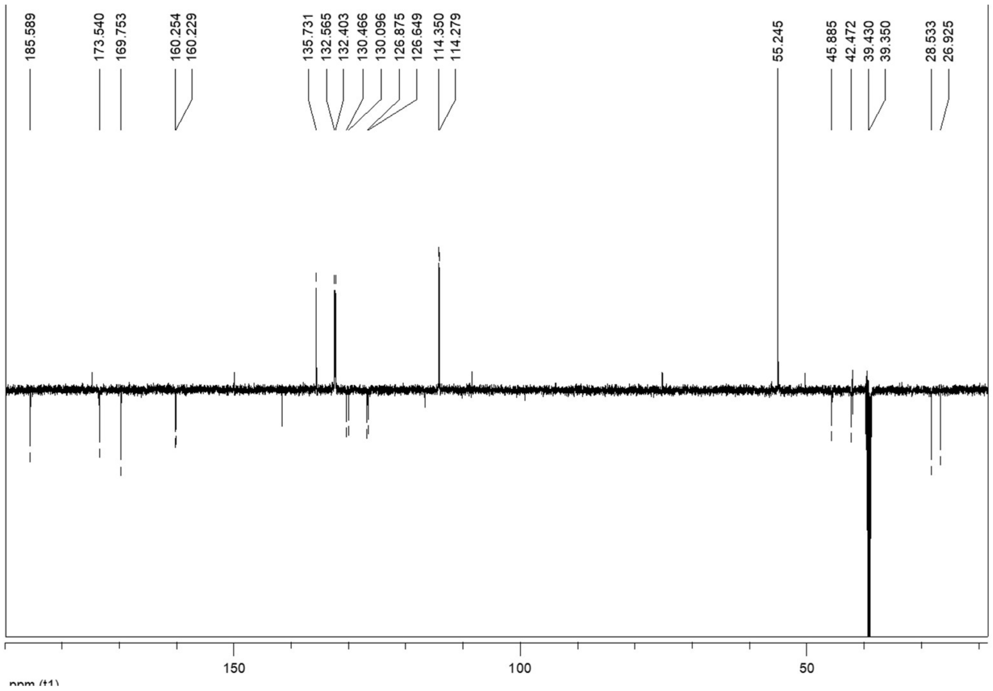
185.589
173.540
169.753
160.254
160.229
135.731
132.565
132.403
130.466
130.096
126.875
126.649
114.350
114.279
55.245
45.885
42.472
39.430
39.350
28.533
26.925
150
100
50

Compound 6:

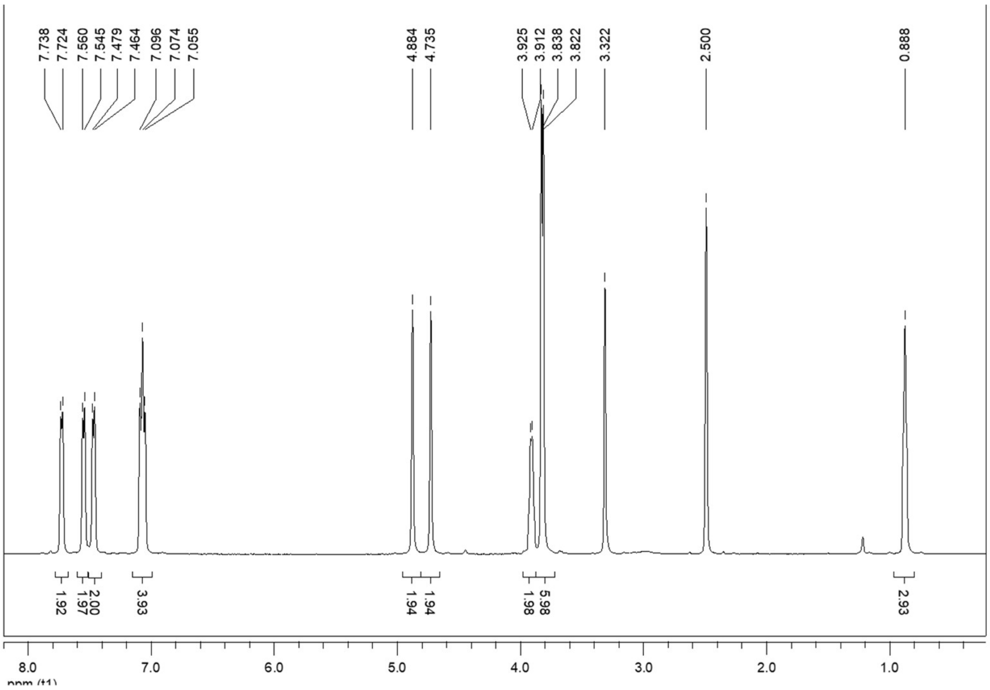
7.738
7.724
7.560
7.545
7.479
7.464
7.096
7.074
7.055
4.884
4.735
3.925
3.912
3.838
3.822
3.322
2.500
0.888
1.92
1.97
2.00
3.93
1.94
1.94
1.98
5.98
2.93
8.0
7.0
6.0
5.0
4.0
3.0
2.0
1.0
ppm (t1)

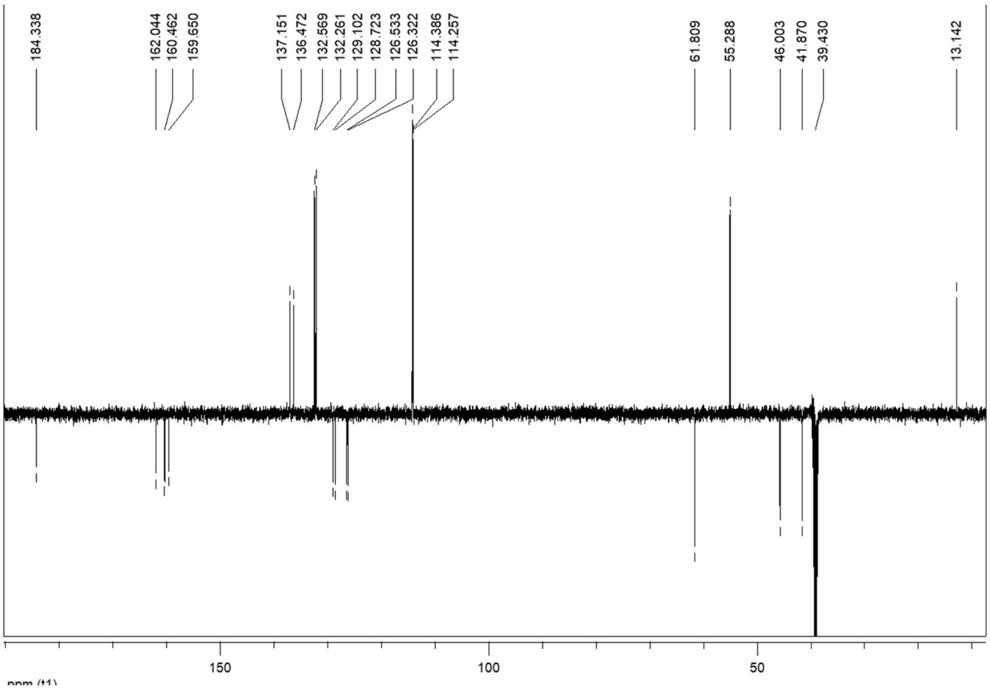
184.338
162.044
160.462
159.650
137.151
136.472
132.569
132.261
129.102
128.723
126.533
126.322
114.386
114.257
61.809
55.288
46.003
41.870
39.430
13.142
150
100
50
ppm (t1)

Compound 7:

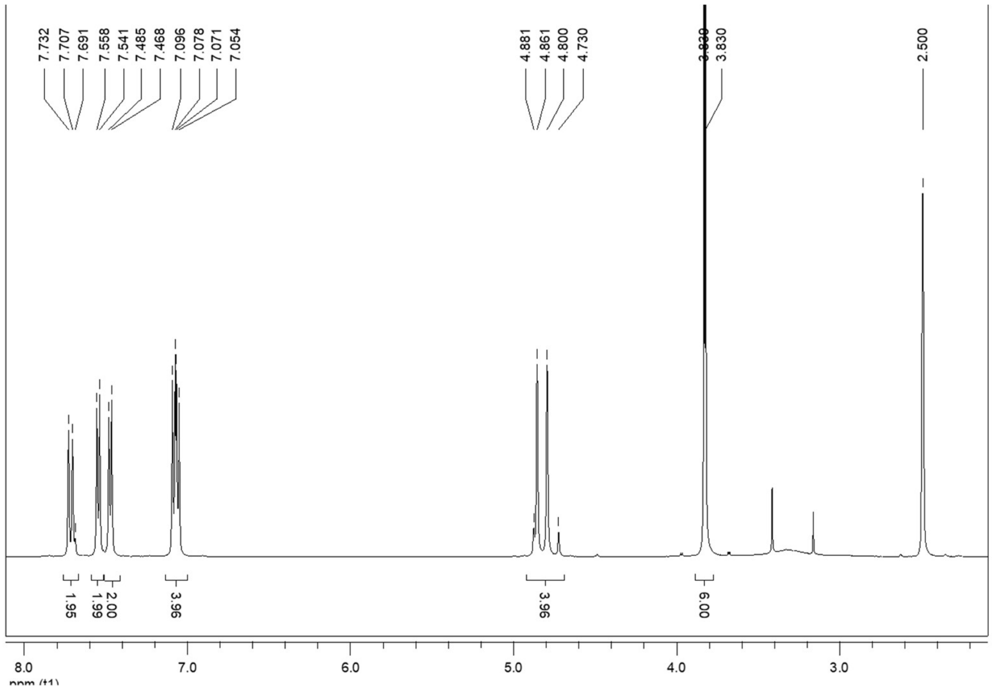
7.732
7.707
7.691
7.558
7.541
7.485
7.468
7.096
7.078
7.071
7.054
4.881
4.861
4.800
4.730
3.830
2.500
1.95
1.99
2.00
3.96
3.96
6.00
8.0
7.0
6.0
5.0
4.0
3.0
ppm (t1)

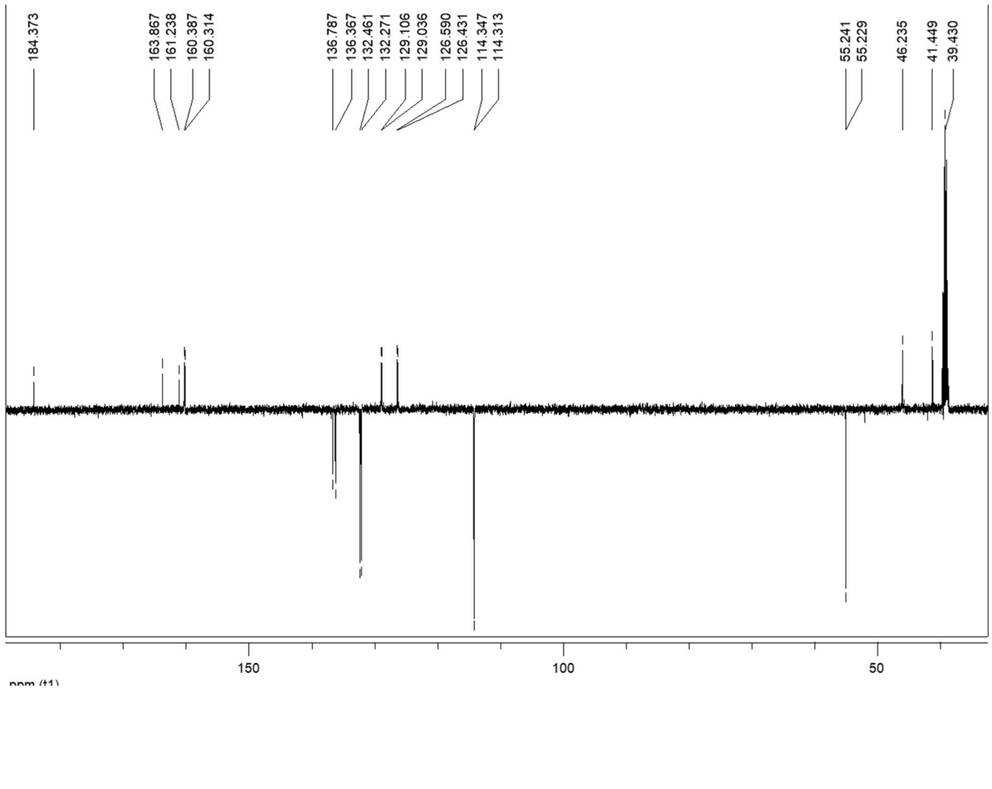
184.373
163.867
161.238
160.387
160.314
136.787
136.367
132.461
132.271
129.106
129.036
126.590
126.431
114.347
114.313
55.241
55.229
46.235
41.449
39.430
150
100
50
ppm (t1)

Compound 8:

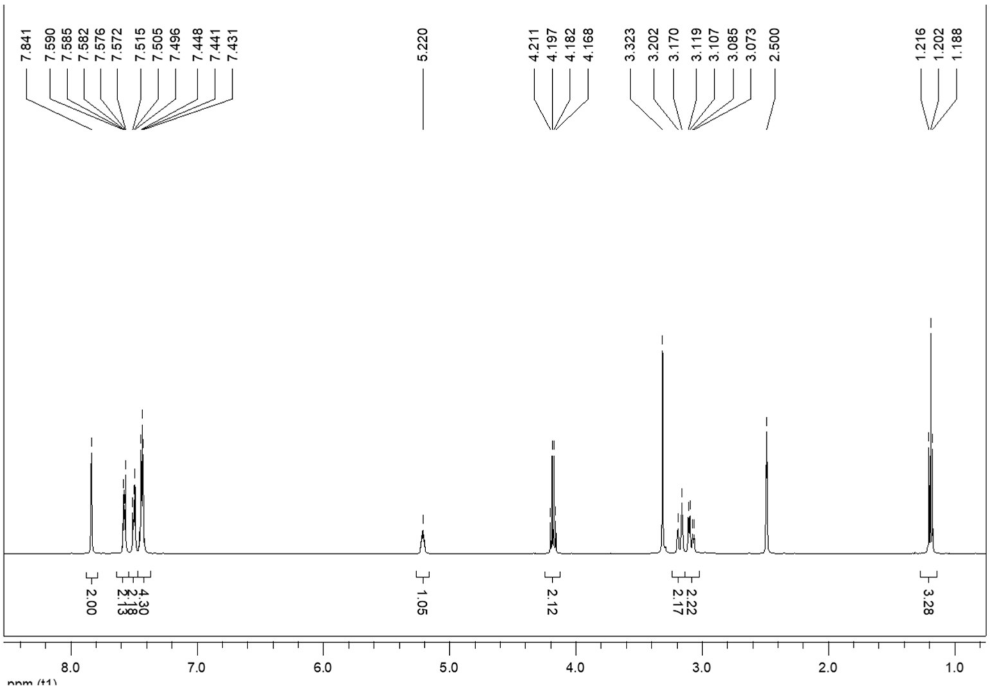
7.841
7.590
7.585
7.582
7.576
7.572
7.515
7.505
7.496
7.448
7.441
7.431
5.220
4.211
4.197
4.182
4.168
3.323
3.202
3.170
3.119
3.107
3.085
3.073
2.500
1.216
1.202
1.188
2.00
2.13
2.18
4.30
1.05
2.12
2.17
2.22
3.28
8.0
7.0
6.0
5.0
4.0
3.0
2.0
1.0
ppm (t1)

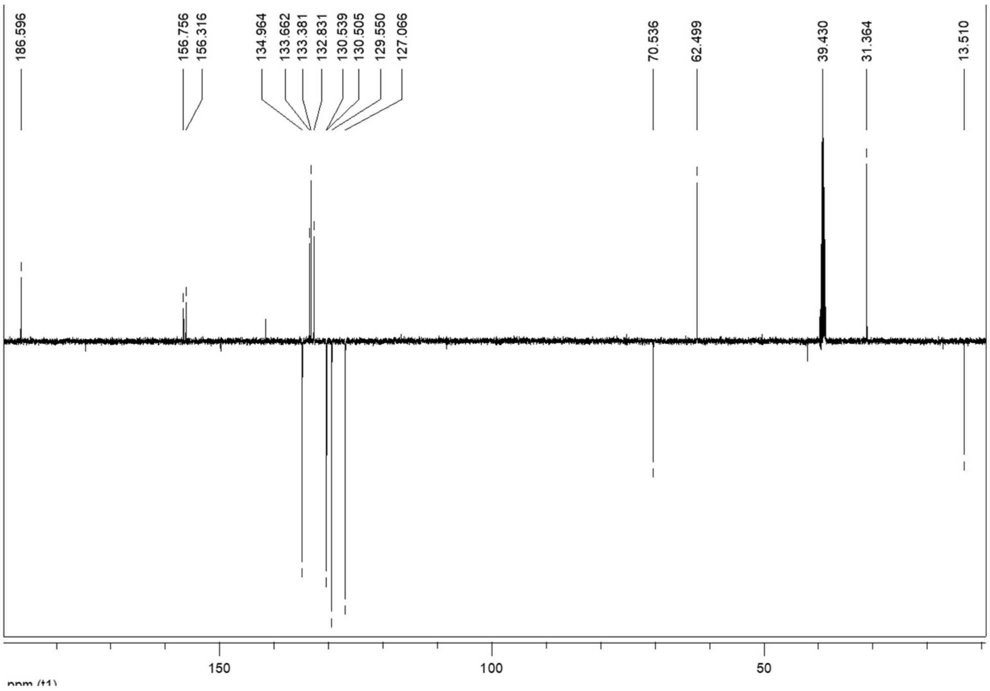
186.596
156.756
156.316
134.964
133.662
133.381
132.831
130.539
130.505
129.550
127.066
70.536
62.499
39.430
31.364
13.510
150
100
50
ppm (t1)

Compound 9:

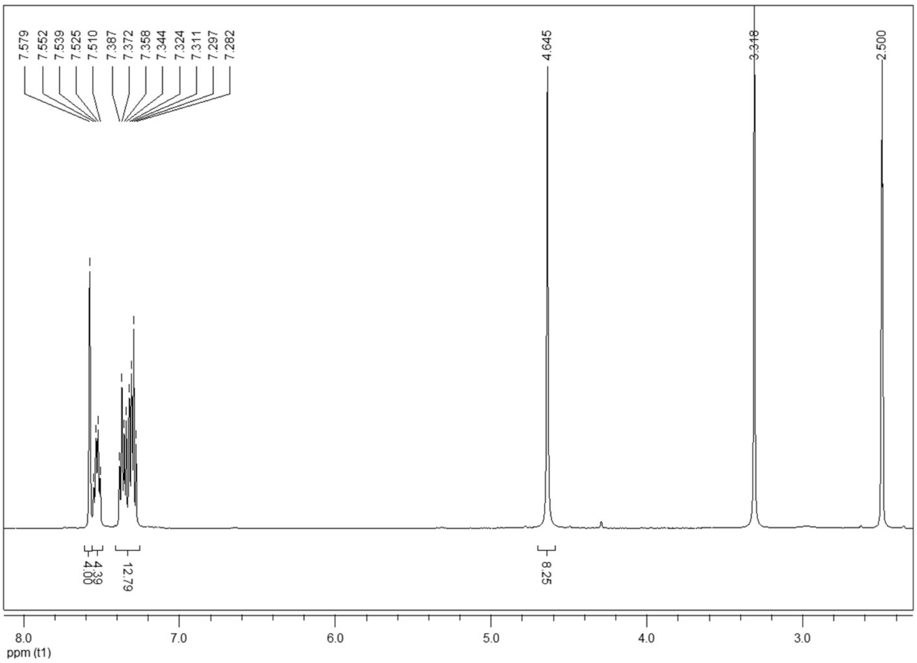

7.579
7.552
7.539
7.525
7.510
7.387
7.372
7.358
7.344
7.324
7.311
7.297
7.282
4.645
3.318
2.500
4.00
4.39
12.79
8.25
8.0
7.0
6.0
5.0
4.0
3.0
ppm (t1)

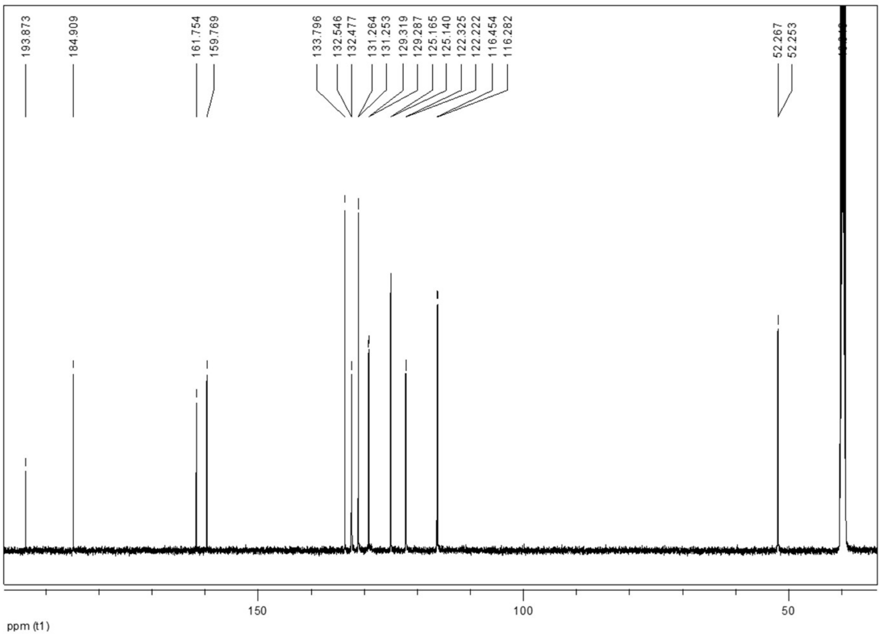

193.873
184.909
161.754
159.769
133.796
132.546
132.477
131.264
131.253
129.319
129.287
125.165
125.140
122.325
122.222
116.454
116.282
52.267
52.253
150
100
50
ppm (t1)
