# Supplementary material for: Novel cyclic C5-curcuminoids possess anticancer activities against HeLa cervix carcinoma, HEC-1A adenocarcinoma, and T24 bladder carcinoma cells
Source: Cancer Cell Int. 2025 Dec 3;25:431. doi: 10.1186/s12935-025-04077-2 (PMC12676898; doi:10.1186/s12935-025-04077-2)

Compound 4:

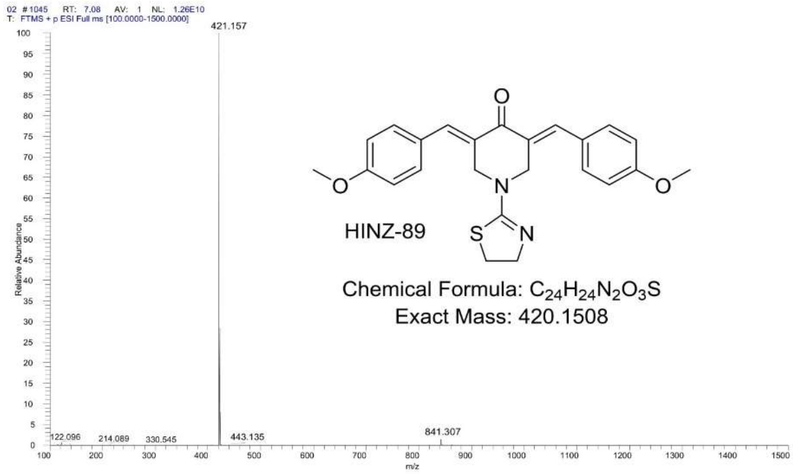

Compound 5:

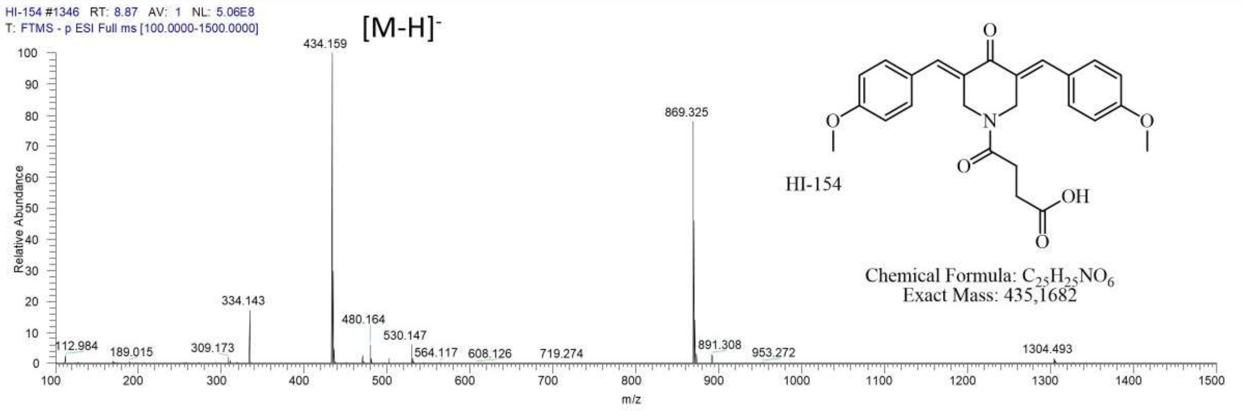

Compound 6:

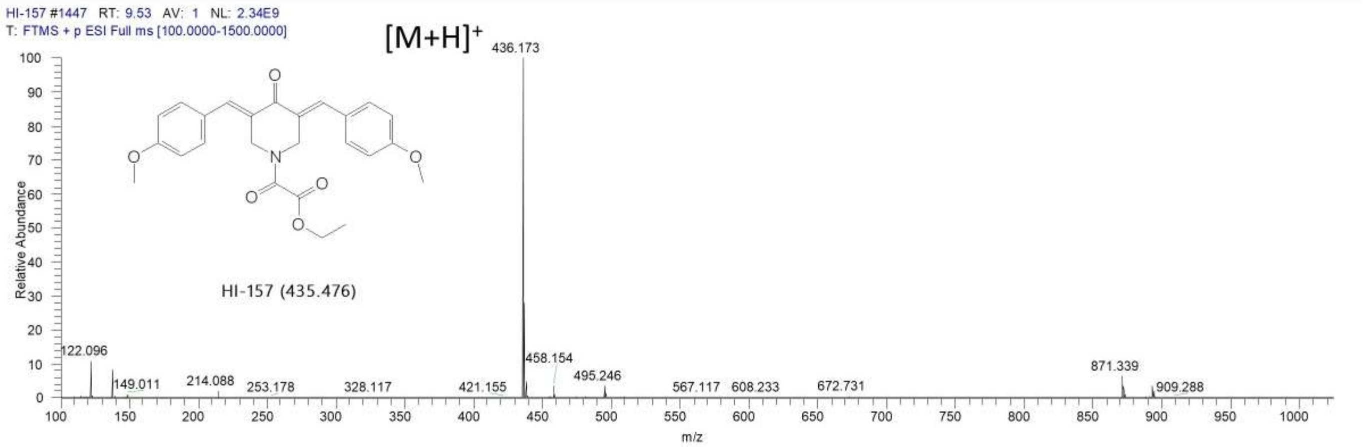

Under the conditions used in this study, all compounds showed the formation of the expected molecular ion peaks (see Section 2.2). However, according to our previous study [30], not only fragments but formation of dimers could be observed. Another general feature is that fragmentation also takes place on the substituent on the central ring (usually on the nitrogen). For example, compound **5** loses the complete acid substituent from the nitrogen atom, resulting in a fragment at 334. At the same time, an unexpected dimer appears at 869, and even a trimer appears at 1304. This dimer formation reaction is a common type of autodimerization in photochemistry. The product of this cycloaddition is a dispiro cyclobutene derivative. This species may be formed via parallel proton/hydride transfer, as previously described [30]. Further examples of dimer formation are: compounds **4** at 841, **6** at 871, **7** at 815, and **9** at 1329. Other examples for fragments from central substituent: compound **8** at 359 or **7** at 364. The appearance of the different product ions also depends on the positive or negative mode used to record the spectra.

Compound 7:

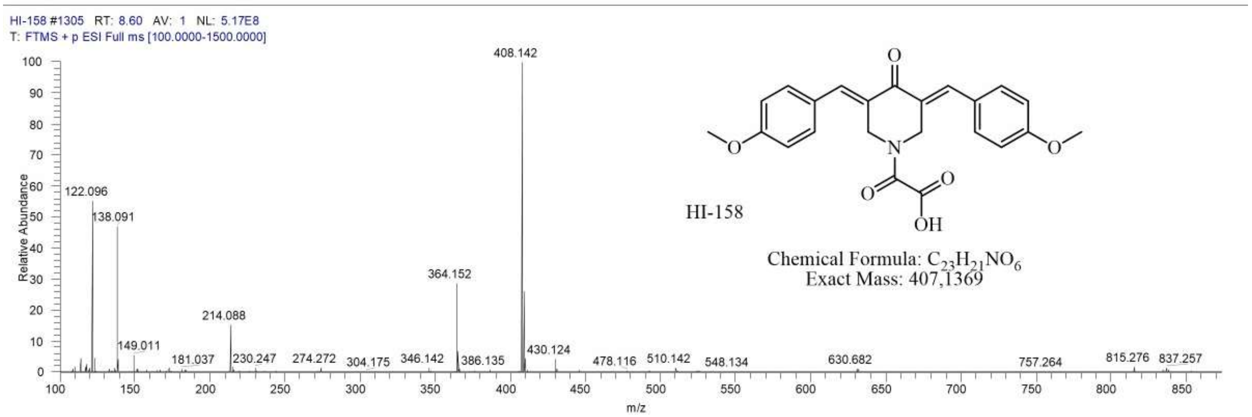

Compound 8:

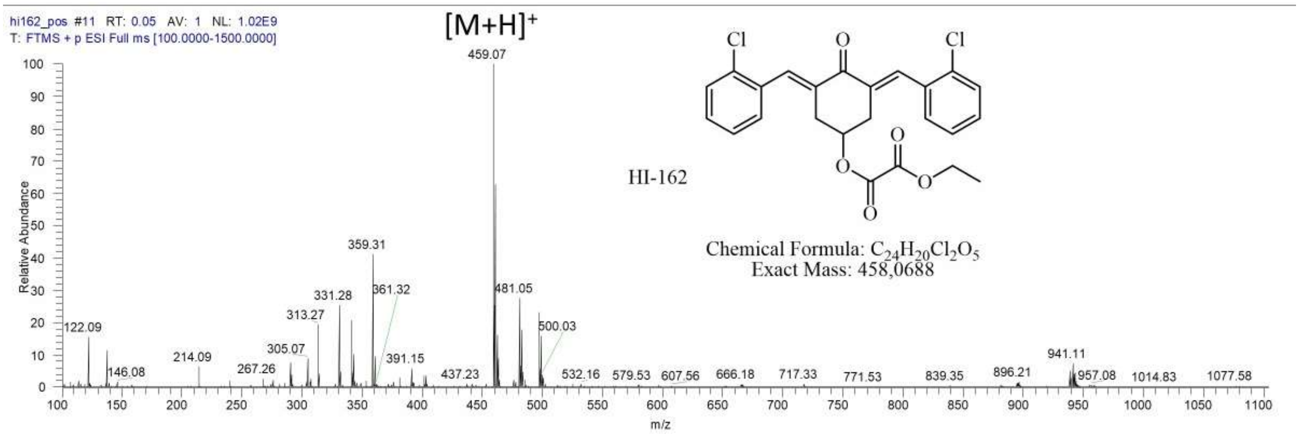

Compound 9:

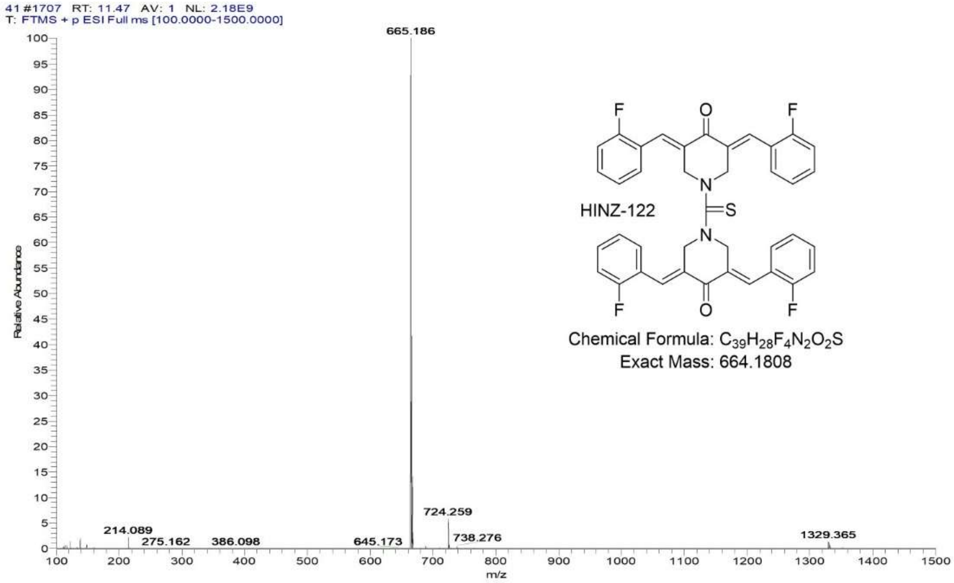

Supplement: Supplementary file 2 — Additional file 2 [file 12935_2025_4077_MOESM2_ESM.pdf]
